# Duration of Protection Against Clinical Malaria Provided by Three Regimens of Intermittent Preventive Treatment in Tanzanian Infants

**DOI:** 10.1371/journal.pone.0009467

**Published:** 2010-03-01

**Authors:** Matthew Cairns, Roly Gosling, Ilona Carneiro, Samwel Gesase, Jacklin F. Mosha, Ramadhan Hashim, Harparkash Kaur, Martha Lemnge, Frank W. Mosha, Brian Greenwood, Daniel Chandramohan

**Affiliations:** 1 Department of Epidemiology and Population Health, London School of Hygiene & Tropical Medicine, London, United Kingdom; 2 Department of Infectious and Tropical Diseases, London School of Hygiene & Tropical Medicine, London, United Kingdom; 3 National Institute for Medical Research, Tanga Centre, Tanga, Tanzania; 4 Kilimanjaro Christian Medical College, Moshi, Tanzania; Walter and Eliza Hall Institute of Medical Research, Australia

## Abstract

**Background:**

Intermittent preventive treatment in infants (IPTi) is a new malaria control tool. However, it is uncertain whether IPTi works mainly through chemoprophylaxis or treatment of existing infections. Understanding the mechanism is essential for development of replacements for sulfadoxine-pyrimethamine (SP) where it is no longer effective. This study investigated how protection against malaria given by SP, chlorproguanil-dapsone (CD) and mefloquine (MQ), varied with time since administration of IPTi.

**Methods and Findings:**

A secondary analysis of data from a randomised, placebo-controlled trial in an area of high antifolate resistance in Tanzania was conducted. IPTi using SP, CD, MQ or placebo was given to 1280 infants at 2, 3 and 9 months of age. Poisson regression with random effects to adjust for potential clustering of malaria episodes within children was used to calculate incidence rate ratios for clinical malaria in defined time strata following IPTi. The short-acting antimalarial CD gave no protection against clinical malaria, whereas long-acting MQ gave two months of substantial protection (protective efficacy (PE) 73.1% (95% CI: 23.9, 90.5) and 73.3% (95% CI: 0, 92.9) in the first and second month respectively). SP gave some protection in the first month after treatment (PE 64.5% (95% CI: 10.6, 85.9)) although it did not reduce the incidence of malaria up to 12 months of age. There was no evidence of either long-term protection or increased risk of malaria for any of the regimens.

**Conclusion:**

Post-treatment chemoprophylaxis appears to be the main mechanism by which IPTi protects children against malaria. Long-acting antimalarials are therefore likely to be the most effective drugs for IPTi, but as monotherapies could be vulnerable to development of drug resistance. Due to concerns about tolerability, the mefloquine formulation used in this study is not suitable for IPTi. Further investigation of combinations of long-acting antimalarials for IPTi is needed.

**Trial Registration:**

Clinicaltrials.gov NCT00158574

## Introduction

Intermittent preventive treatment in infants (IPTi) is a new control strategy for reducing the malaria burden in endemic countries of sub-Saharan Africa where transmission of malaria is high and malaria is an important cause of mortality and morbidity in infants. There is now sufficient evidence that three courses of sulfadoxine-pyrimethamine (SP) IPTi has a protective efficacy of 20–30% against clinical malaria in the first year of life; therefore SP-IPTi may be adopted as a policy in some African countries [1], [2]. However, widespread and escalating resistance to SP remains a concern and it is unlikely that SP-IPTi will remain effective in areas where there is high prevalence of SP resistance, as has been the case for IPT in pregnant women [3], [4]. Consequently, other drugs need to be developed urgently for IPTi.

Although the first IPTi study found a suggestion of sustained protection [5], [6], subsequent SP-IPTi trials have established that in most circumstances, protection is likely to be attributable to the direct effect of the drugs used [7]–[11]. Nevertheless, some uncertainty about the precise mode of action of IPTi remains, in particular, the relative importance of clearing existing parasitaemia compared to post-treatment prophylaxis against new infections is not certain, and may vary in different epidemiological settings [12]. Two studies of the duration of protection against malaria after IPTi using SP have suggested that post-treatment prophylaxis may be the main protective mechanism [13], [14], but this is not yet certain, and it is unclear whether this will be the case for IPTi regimens other than SP.

To investigate these issues, an individually randomised, placebo-controlled trial of SP and two alternative regimens (chlorproguanil-dapsone, CD and mefloquine, MQ) was undertaken in an area of high SP resistance in Tanzania [15]. This trial sought to identify whether SP retained its efficacy for IPTi despite a high prevalence of antifolate resistance, whether a short-acting antimalarial such as CD (terminal elimination half-life <40 hours [16]) could be used effectively for IPTi and whether a long-acting antimalarial such as MQ (half-life 2–3 weeks [17]) would be more effective. The trial found that neither SP nor CD provided any protection either during the intervention period (2–11 months) or between 12 and 23 months of age. MQ was found to provide a high level of protection between the ages 2–11 months (PE 38.1% (95% CI 11.8, 56.5), but concerns about its tolerability in infants may make it unsuitable for IPTi in its current formulation.

It could be informative to investigate how protective efficacy in the above study varies with time since treatment. While it is possible that the finding of no benefit overall for SP or CD could mean that there is genuinely no benefit at any time, it is also possible that there is either a small benefit initially after treatment which is too small to remain detectable over the whole of infancy. Alternatively, it may be that an initial benefit of treatment is cancelled out by a subsequent increased risk of clinical malaria, either through loss of premunition [18], [19], or due to suppressed drug resistant parasites surviving treatment and recrudescing at a later stage [20].

The purpose of the present study was therefore to investigate how the protective efficacy of the three drug regimens used in the above trial changed with time since treatment, and thus clarify the mechanism of protection given by IPTi.

## Methods

### Trial Background

This study uses data from the Kilimanjaro IPTi Drug Options Trial, described in detail elsewhere [15]. The protocol for this trial is available as supporting information; see Protocol S1. The trial was registered as a randomised clinical trial with the National Institute for Health clinical trials registry (www.clinicaltrials.gov identifier: NCT00158574), and was approved by the Ethics Review Board of the National Institute for Medical Research of Tanzania and the London School of Hygiene and Tropical Medicine. Briefly, a double-blind, individually randomised, placebo-controlled trial of IPTi using SP, CD and MQ was undertaken in two districts in Tanzania with a high prevalence of SP resistance. Full treatment doses of the study drugs were given to children enrolled in the trial at the time of the second diphtheria-pertussis-tetanus/polio vaccine (DPT/polio 2), DPT/polio 3 and measles vaccinations at approximately 2, 3 and 9 months of age. The separate IPT rounds are referred to in this paper as IPT1, IPT2 and IPT3. Children were followed-up until two years of age for incidence of the primary outcome of clinical malaria (temperature ≥37.5°C or history of fever within 48 hours plus parasitaemia of any density).

The analyses presented here use only data from the moderate transmission site in Korogwe, Tanga Region, Tanzania. Bødker *et al*. [21] reported an entomological inoculation rate (EIR) of nearly 100 infectious bites per year in the Korogwe lowlands, though it is thought that transmission has since declined as it has in other regions of Tanzania [22] and South and East Africa [23]. Recently, an *in vivo* efficacy study of SP in this region showed a day 28 adequate clinical and parasitological response of only 18% [24].

### Data Analysis

Children who had received and successfully swallowed a given round of IPT (IPT1, IPT2 or IPT3) were included in the analysis. The endpoint was clinical malaria, as defined above, detected passively at one of the study clinics. Time at risk was calculated from time of IPT treatment until completion of five months (150 days) of follow-up, receipt of a subsequent dose of IPT or exit from the study (emigration, death, refusal or exclusion), whichever occurred first. As in the main trial analysis, children who had a malaria episode or who were treated with an antimalarial had 21 days removed from the person time at risk. To enable month specific estimates of the protective efficacy by time since treatment, follow-up time was stratified by month (30 days) since treatment using lexis expansion [25].

Poisson regression was used to calculate incidence rate ratios for clinical malaria, allowing multiple episodes of malaria per child. To adjust for potential clustering of malaria episodes within children, Poisson regression models with gamma distributed random effects were used. Multivariate models were built using an additive step-wise approach, using the likelihood ratio test to compare models and aiming for the simplest model that adequately explained the data. Since the principal interest of this study was the interaction between intervention group and time since treatment, this interaction was modelled first. Covariates known to be associated with the incidence of malaria in this population from the main trial were then added to the models (ownership of an insecticide treated net, rural residence and distance of residence from the nearest health facility).

To explore the changes in protective efficacy against clinical malaria over time, malaria incidence rate ratios for SP, CD and MQ versus placebo by month since IPT were calculated. Protective efficacy and its 95% confidence interval was then calculated as 1-rate ratio. To obtain more precise estimates of protective efficacy over time, a summary analysis was then performed combining time at risk and malaria episodes following all three IPT rounds into a single analysis that related malaria incidence to time since most recent IPT. To account for possible differences in efficacy between separate IPT rounds, the IPT round from which time at risk and incidence was taken was tested for inclusion in the multivariate regression models as an interaction term with treatment group and as a covariate.

## Results

In the analysis of the clinical trial, IPTi with MQ was shown to provide a protective efficacy against clinical malaria of 38.1% (95% CI 11.8, 56.5) in infants aged 2–11 months of age. There was no statistically significant protective effect from SP −6.7% (−45.9, 22.0) or CD 10.8% (−24.6, 36.1) during infancy [15].

It was not possible to calculate protective efficacy by month after IPT1, since most children received IPT2 one month later (75% within 28 days and 95% within 42 days of IPT1) and incidence of malaria in the placebo group in this period was very low (table 1). Inclusion of treatment round number in the regression models did not improve the fit of the model to the data for the multi-round analyses of either SP or CD but it was included as a covariate in the model for the multi-round analysis of mefloquine protective efficacy.

**Table 1:** Malaria incidence rate by month after IPTi.

		IPT1			IPT2			IPT3			All IPT rounds		
	Month	Cases	Rate	95 % CI	Cases	Rate	95 % CI	Cases	Rate	95 % CI	Cases	Rate	95 % CI
Placebo	1	1	0.04	(0.01, 0.29)	2	0.08	(0.02, 0.32)	14	0.62	(0.37, 1.05)	17	0.24	(0.14, 0.38)
	2	1	0.82	(0.12, 5.84)	3	0.12	(0.04, 0.38)	6	0.27	(0.12, 0.6)	10	0.21	(0.1, 0.38)
	3	1	1.67	(0.23, 11.7)	12	0.50	(0.28, 0.87)	9	0.41	(0.21, 0.78)	22	0.47	(0.29, 0.71)
	4	0	0	.	12	0.50	(0.28, 0.88)	7	0.31	(0.15, 0.65)	19	0.40	(0.24, 0.63)
	5	0	0	.	8	0.33	(0.16, 0.66)	9	0.41	(0.21, 0.78)	17	0.36	(0.21, 0.58)
SP	1	3	0.12	(0.04, 0.38)	2	0.08	(0.02, 0.32)	2	0.09	(0.02, 0.35)	7	0.10	(0.04, 0.2)
	2	1	0.74	(0.1, 5.29)	9	0.36	(0.19, 0.7)	9	0.40	(0.21, 0.77)	19	0.39	(0.24, 0.61)
	3	0	0	.	7	0.29	(0.14, 0.61)	10	0.45	(0.24, 0.83)	17	0.36	(0.21, 0.57)
	4	0	0	.	19	0.79	(0.51, 1.25)	15	0.68	(0.41, 1.13)	34	0.73	(0.51, 1.02)
	5	0	0	.	10	0.42	(0.23, 0.79)	6	0.27	(0.12, 0.6)	16	0.35	(0.2, 0.56)
CD	1	4	0.17	(0.06, 0.46)	2	0.08	(0.02, 0.33)	6	0.28	(0.13, 0.63)	12	0.17	(0.09, 0.3)
	2	0	0	.	3	0.13	(0.04, 0.39)	6	0.29	(0.13, 0.63)	9	0.19	(0.09, 0.37)
	3	0	0	.	5	0.21	(0.09, 0.5)	4	0.19	(0.07, 0.5)	9	0.20	(0.09, 0.37)
	4	0	0	.	9	0.39	(0.2, 0.75)	5	0.24	(0.1, 0.57)	14	0.31	(0.17, 0.52)
	5	1	0.95	(0.13, 6.77)	9	0.39	(0.2, 0.75)	5	0.24	(0.1, 0.57)	15	0.33	(0.19, 0.55)
MQ	1	1	0.04	(0.01, 0.31)	1	0.04	(0.01, 0.3)	3	0.14	(0.05, 0.43)	5	0.07	(0.02, 0.17)
	2	0	0	.	1	0.04	(0.01, 0.3)	2	0.09	(0.02, 0.38)	3	0.06	(0.01, 0.19)
	3	0	0	.	9	0.39	(0.2, 0.75)	6	0.28	(0.13, 0.63)	15	0.33	(0.18, 0.54)
	4	0	0	.	12	0.53	(0.3, 0.93)	12	0.58	(0.33, 1.02)	24	0.53	(0.34, 0.79)
	5	0	0	.	3	0.13	(0.04, 0.4)	12	0.58	(0.33, 1.03)	15	0.33	(0.19, 0.55)

Malaria incidence rate per person year and 95% confidence interval by month following treatment for IPT doses 1–3 and all IPT rounds combined. Most children exited follow-up after the administration of IPT1 around one month later, when they received IPT2 at approximately 3 months of age. Abbreviations: CI, confidence interval; SP, sulfadoxine-pyrimethamine; CD, chlorproguanil-dapsone; MQ, mefloquine.

### Sulfadoxine-Pyrimethamine

**Figure 1:**
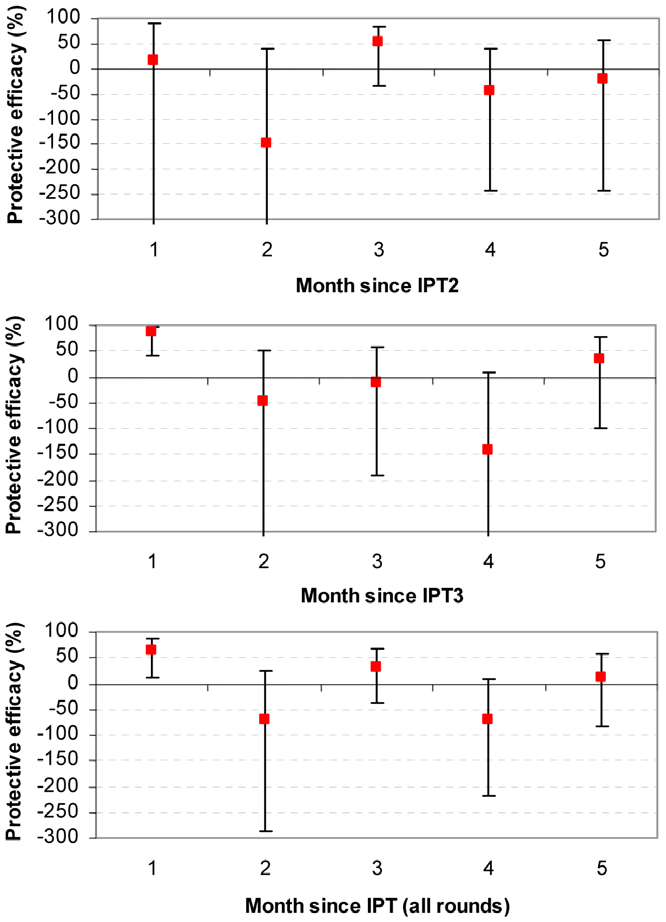
Protective efficacy of SP against clinical malaria. Protective efficacy (1- rate ratio vs. placebo) is shown by month since treatment. Y-axes for IPT2 and IPT3 graphs are truncated at -300, for full extent of confidence interval see table 2.

There was no statistically significant evidence that SP gave any protection in any specific month after IPT2 (Table 2 and Figure 1). However, the confidence intervals around these estimates are wide, reflecting the low incidence rate in these young children. There was statistically significant evidence of considerable protection in the first month after IPT3 (protective efficacy (PE) 87.5% (95% CI: 42.4, 97.3); p = 0.008) but no further protection after this time. In the summary analysis of all rounds of IPT combined, there was again evidence of protection in the first month after treatment, (PE 64.5% (10.6, 85.9); p = 0.028), but no evidence of protection beyond this time. There was a suggestion of increased incidence in children given SP compared to those given placebo in some months, but this was not statistically significant.

**Table 2:** Protective efficacy against clinical malaria by month after IPTi.

		IPT2	IPT3	All IPT rounds
	Month	Protective efficacy (95% CI)	Protective efficacy (95% CI)	Protective efficacy (95% CI)
SP	1	17 (−524.1, 89)	87.5 (42.4, 97.3)	64.5 (10.6, 85.9)
	2	−148.7 (−900.7, 38.2)	−47.8 (−340.8, 50.4)	−70.8 (−285.5, 24.4)
	3	52.7 (−35.4, 83.4)	−10 (−190.6, 58.4)	31.8 (−36.2, 65.8)
	4	−44.7 (−243.9, 39.1)	−143 (−541, 7.9)	−70.9 (−219.6, 8.6)
	5	−21 (−244.4, 57.5)	33 (−100.5, 77.6)	12.6 (−82.7, 58.2)
CD	1	8.2 (−591.9, 87.8)	54.5 (−25.6, 83.5)	28.9 (−55.5, 67.5)
	2	8 (−391.3, 82.8)	−18.3 (−286.8, 63.8)	8 (−134.9, 63.9)
	3	67.4 (−3.6, 89.7)	48.6 (−75.3, 84.9)	59.4 (8, 82)
	4	34.8 (−76, 75.8)	14.3 (−183.5, 74.1)	23.5 (−59.6, 63.4)
	5	−20.8 (−251, 58.4)	39.9 (−89.4, 80.9)	7.5 (−94, 55.9)
MQ	1	56.5 (−415.5, 96.3)	80.1 (27.6, 94.5)	73.1 (23.9, 90.5)
	2	72.2 (−188.8, 97.3)	69.7 (−55.2, 94.1)	73.3 (0, 92.9)
	3	33.1 (−92.4, 76.7)	34.2 (−94.4, 77.7)	36.1 (−30.9, 68.8)
	4	10.7 (−141.5, 67)	−88 (−405.8, 30.1)	−25.6 (−144.7, 35.5)
	5	63.1 (−58, 91.4)	−38.1 (−248.7, 45.3)	13.7 (−83, 59.3)

Estimates of protective efficacy against clinical malaria, calculated as (1- rate ratio of active treatment [SP, CD or MQ] vs. placebo). All regression models were adjusted for the covariates ITN use, rural residence and distance from nearest health facility. Mefloquine (All IPT rounds) was also adjusted for IPT round. Most children exited follow-up after the administration of IPT1 around one month later, when they received IPT2 at approximately 3 months of age. For this reason this analysis was not possible for follow-up after IPT1. Abbreviations: CI, confidence interval; SP, sulfadoxine-pyrimethamine; CD, chlorproguanil-dapsone; MQ, mefloquine.

### Chlorproguanil-Dapsone

**Figure 2:**
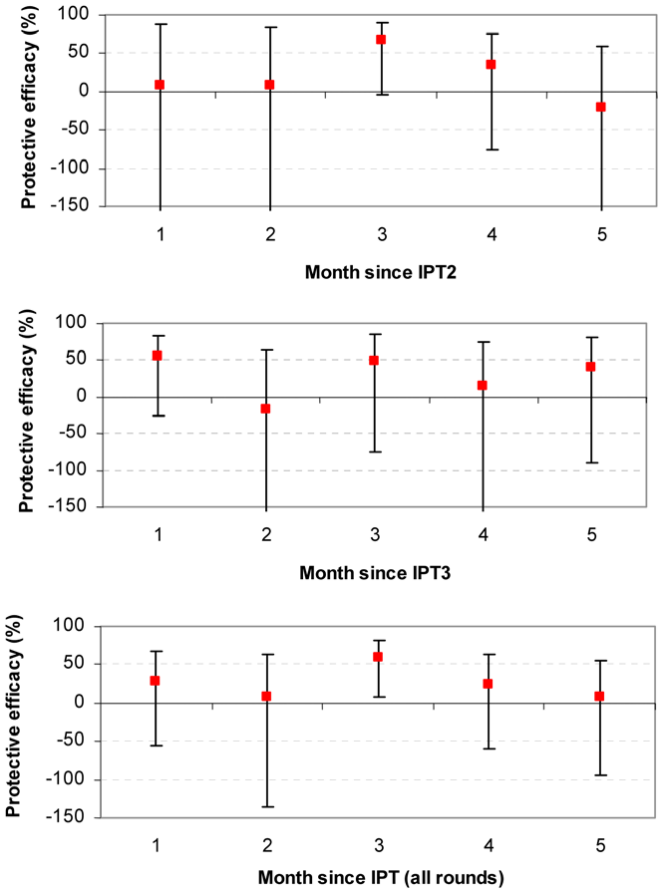
Protective efficacy of CD against clinical malaria. Protective efficacy (1- rate ratio vs. placebo) is shown by month since treatment. Y-axes for IPT2 and IPT3 graphs are truncated at -150, for full extent of confidence interval see table 2.

There was no evidence of protection from CD in most months after treatment (Table 2 and Figure 2). There was a suggestion that CD gave some protection during the third month after IPT2 (PE 67.4% (−3.6, 89.7); p = 0.057) and in the third month in the summary analysis of all 3 IPT rounds (PE 59.4% (8.0, 82.0); p = 0.031). Consistent with the finding during the whole trial period, there was no evidence of any protective benefit of IPT with CD at any other time.

### Mefloquine

**Figure 3:**
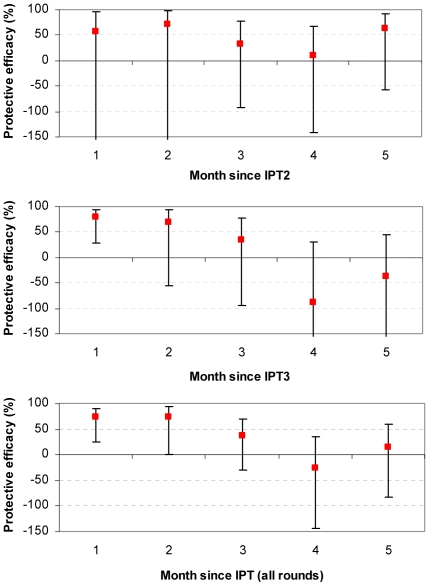
Protective efficacy of MQ against clinical malaria. Protective efficacy (1- rate ratio vs. placebo) is shown by month since treatment. Y-axes for IPT2 and IPT3 graphs are truncated at -150, for full extent of confidence interval see table 2.

The point estimates of protective efficacy suggested some protection in the two months following IPT2, but the confidence interval overlaps unity (Table 2 and Figure 3). Beyond the 2 month period there was no evidence of any protection or any detrimental effect of IPT2. There was strong evidence of protection in the first month after IPT3 (PE 80.1% (27.3, 94.5); p = 0.014, and suggestion of protection in the second month that was not statistically significant (PE 69.7% (−55.2, 94.1), p = 0.152).

In the summary analysis of all 3 IPT rounds, there was strong evidence of a high level of protection in the first month (PE 73.1% (23.9, 90.5); p = 0.013). The point estimate of protective efficacy was similarly high in the second month, but this was only of borderline statistical significance (PE 73.3% (0, 92.9); p = 0.050). There was a suggestion of protection in the third month but this was not statistically significant. There is thus no strong evidence of any benefit or detrimental effect in months three, four or five after IPT with MQ.

## Discussion

### Principal Findings

Mefloquine provided a high level of protection against clinical malaria in the first two months after IPTi, consistent with its extended plasma half-life and its presumed therapeutic efficacy in Africa [17], [26]. There was no increased risk of malaria once protection from MQ had waned, suggesting that there is no detrimental effect in the post-IPT period.

SP provided some protection against clinical malaria in the first month after IPT, despite the high prevalence of antifolate resistance in the study site [24]. There was no statistically significant evidence of either a protective or detrimental effect in any period beyond the first month, though the point estimate and confidence interval in the second month were consistent with a modest detrimental effect.

Chlorproguanil-dapsone provided little protection against clinical malaria compared to placebo. While there was a suggestion of some protection in the third month after IPT, largely due to apparent protection after IPT2, we consider this likely to be a chance finding given the borderline statistical significance and the lack of a plausible biological mechanism for protection to be delayed to such a specific and finite window after treatment. Chlorproguanil-dapsone was shown to be effective in treating acute malaria episodes that had failed treatment with SP in Tanzania [27] and SP-resistance was associated with failure of treatment with SP but not CD in Malawi [28]. It therefore appears likely that the poor performance of CD as an IPT regimen is due to its short half-life.

### Limitations

Due to the relatively low incidence rate of clinical malaria in this study population, estimates of the intervention effect are less precise than would be ideal for this type of analysis, and the time-stratified analyses are likely to be underpowered to detect effects in specific time strata. This is particularly the case when examining the protective efficacy of IPT1 and IPT2. The low incidence rate also limits the minimum size of time strata for which sensibly precise estimates of protective efficacy can be calculated to around one month. The analytical approach includes multiple comparisons and thus it is possible some spurious low p-values may have appeared due to chance. However, in the present analysis, the directions of trends can still be seen, which combined with understanding of the pharmacodynamics of the antimalarials allows a biologically plausible interpretation to be made. With a larger trial or a higher incidence rate of malaria, it would have been possible to stratify follow-up more finely to get a more detailed picture of how protection varies over time, as was possible in a similar analysis of the Navrongo IPTi trial [13]. It would also be possible to set lower thresholds for statistical significance in order to better exclude the role of chance.

To assess if our findings could have been affected by the decision to stratify follow-up time by month, the analyses were repeated with different sized time strata. This did not alter the estimate of duration of protection or the interpretation (data not shown). We did not find any evidence of interaction between IPTi drug group and IPTi round (i.e. there was no suggestion that efficacy of IPTi compared to placebo varied between the different rounds), but it would be preferable to have greater precision to look at separate rounds in isolation.

### Interpretation

Short-acting antimalarials are unlikely to be a suitable choice for intermittent preventive treatment in infants. Longer-acting antimalarials with a duration of action similar to that of mefloquine and SP are likely to be more efficacious, since they would provide a substantial period of post-treatment prophylaxis. However, the mefloquine formulation used in this trial is not likely to be suitable for IPTi because it is poorly tolerated [15], and the usefulness of SP in areas where resistance is high may be severely limited. The protective efficacy of SP within the first month seen here (64.5% (10.6, 85.9)) is comparable with that seen in a previous study in Ghana, where protection against malaria in the first month was 75.2% (66, 82) [13], but unlike in Ghana, after one month there was no evidence of any further protection, and no overall benefit of IPTi in infancy [15].

The finding of protection limited to a shorter period immediately after IPTi with SP suggests that malaria episodes are only prevented when plasma concentrations of both sulfadoxine and pyrimethamine are greatest and the two components act in synergy; this period may last for 15 days for antifolate resistant parasites [29]. Shortened post-treatment prophylaxis is known to be a consequence of drug resistance [30], and indeed protection from SP was shorter than in other studies of SP-IPTi in areas with lower SP resistance [13], [14].The suggestion of slightly increased incidence of malaria in the second month after SP-IPTi, combined with our knowledge of resistance patterns in the study site and the overall lack of any protection in infancy overall is consistent with the hypothesis that SP was not able to completely eliminate highly resistant parasites. We speculate that when drug levels waned sufficiently, previously suppressed infections then increased in density and caused clinical attacks of malaria. Measuring protective efficacy in a short period after an IPTi course (e.g. the first month) could be misleading since it would miss recrudescence that was delayed beyond this time by a long-acting but ultimately ineffective antimalarial. Similar arguments have previously been made for assessment of therapeutic efficacy [31].

The overall protective efficacy of mefloquine up to 12 months of age in this trial was 38.1% (95% CI: 11.8, 56.5), which compares favourably with most of the trials of SP [7]–[11]. Protection appears to be concentrated in the first two months after the dose, making the duration of protection slightly longer than in infants given SP [13], [14]. Since mefloquine has a longer half-life than SP (2 to 3 weeks versus 7 days for sulfadoxine and 3 days for pyrimethamine [17], [20], [29], [32]), a slightly longer period of post-treatment prophylaxis would be expected. In a separate study of the efficacy of mefloquine for the treatment of asymptomatic parasitaemia completed in 24 month old children, we found that just below half of 24 month old children still had detectable levels of mefloquine 56 days after treatment (data not shown). These findings are therefore coherent, and consistent with the idea that the benefits of IPTi are restricted to the direct pharmacodynamics of the drugs involved.

If long-acting drugs offer the best protection when used for IPTi, this raises an immediate dilemma. Slowly eliminated antimalarial drugs may be most prone to development of drug resistance [33]. It is not clear how this problem can best be tackled. One solution could be the combination of long acting drugs with similar pharmacokinetic profiles since these could protect each other from selection of resistant genotypes as they are progressively eliminated [34]. However, this has not been adequately investigated in practice, and there are few candidate antimalarials currently available for this role. Single dose regimens for IPT would be preferable to a course given over several days. Safety will be a particularly important consideration when developing future IPTi regimens, since many children who receive IPT will be healthy at the time they are treated. Other studies undertaken by the IPTi Consortium will report on the safety profile of the existing IPTi drugs.

### Conclusion

Mefloquine, the most efficacious regimen in this IPTi trial, protected children by giving a substantial period of post-treatment prophylaxis. SP provided protection against clinical malaria for approximately one month, but it appears that some malaria attacks were simply delayed until the following month once drug concentrations had waned. Given concerns about possible side-effects and tolerability of mefloquine, and exacerbation of drug resistance, novel combinations of long-acting antimalarials should be investigated for use in IPTi.

## Supporting Information

Protocol S1 Trial Protocol
(1.08 MB DOC)
