# Supplementary material for: Duration of Protection Against Clinical Malaria Provided by Three Regimens of Intermittent Preventive Treatment in Tanzanian Infants
Source: PLoS One. 2010 Mar 1;5(3):e9467. doi: 10.1371/journal.pone.0009467 (PMC2830887; doi:10.1371/journal.pone.0009467)
Supplement: Protocol S1 — Trial Protocol (1.08 MB DOC) [file pone.0009467.s001.doc]

**Drug options for intermittent preventive treatment for malaria in infants in an area with high resistance to sulfadoxine/pyrimethamine:**

**an evaluation of short and long-acting antimalarial drugs**

**Principal Investigators**

Dr Daniel Chandramohan^1^

Dr Martha Lemnge^2^

Dr Frank Mosha^3^

Prof. Brian Greenwood^1^

**Collaborators**

Dr Julius Massaga^2^

Dr Theonest Mutabingwa^1,2^

Dr Alex Mwita^4^

Dr Ilona Carneiro^1^

Dr Anita Ronn^5^

Dr Hugh Reyburn^1,6^

Dr Christopher Whitty^1^

Dr Roly Gosling^1^

Dr Lasse Vestergaard^5^

Dr Thor Theander^5^

**______________________________________________**

^1^London School of Hygiene & Tropical Medicine, London, UK

^2^National Institute of Medical Research, Amani, Tanzania

^3^Kilimanjaro Christian Medical Centre, Moshi, Tanzania

^4^Malaria Control Programme, Ministry of Health, Tanzania

^5^University of Copenhagen, Denmark

^6^MRC Malaria Research Centre, Moshi, Tanzania

**Protocol version 6: Wednesday, 25 July 2007**

# Table of contents

- List of Abbreviations …………………………………………. 3
- Summary …………………………………………………. 4

1. Background

1.1. Malaria as a cause of anaemia …………………………………. 5

1.2. Prevention of malaria by chemoprophylaxis or IPTi…………… 5

1.3. The mode of action of drugs used for IPTi ………………….. 6

1.4. Choice of drugs for IPTi ………………………………….. 7

1.5. Potential effect of IPTi on clinical immunity ……………………. 10

1.6 The influence of G6PD deficiency and human haemoglobinopathies

10

2. Objectives ………………………………………………….. 11

3. Study area ………………………………………………….. 11

3.1. Change of study site ……………………………………………. 14

3.2 Addition of Hale Dispensary as study site in Lwengera Valley… 14

4. Study methodology

4.1. Study design ………………………………………………….. 14

4.2. Study population: inclusion/exclusion criteria ………………….. 15

4.3. Outcome measures ………………………………………….. 17

4.4. Sample size ………………………………………………….. 17

4.5. Screening, enrolment and follow up procedures ………….. 21

4.6. Randomisation and allocation of study groups ………….. 22

4.7. Dose schedule and administration of study drugs ………….. 22

4.8. Monitoring compliance and safety ………………………….. 23

4.9. Surveillance of clinical malaria ………………………….. 24

4.10. Iron status …………………………………………………… 25

4.11. Evaluation of resistance to study drugs ………………….. 25

4.12. Laboratory procedures ………………………………….. 26

5. Monitoring the trial …………………………………………… 27

6. Data management and analysis …………………………… 27

7. Dissemination of results…………………………………………… 28

8. Ethical considerations …………………………………………… 28

9. Budget …………………………………………………………… 29

10. Time Table …………………………………………………… 32

11. References …………………………………………………… 33

Appendix 1: A deterministic model of the effect of IPTi on anaemia 36

Appendix 2: A comparative study of the efficacy of two or three

courses of antimalarial for IPTi in low transmission areas 38

Appendix 3: Adverse events …………………………………… 39

Appendix 4: Blister pack of drugs for IPTi course 1 & 2 …………….. 41

Appendix 4: Blister pack of drugs for IPTi course 3 ….. …………….. 42

**List of abbreviations**

AIDS Acquired Immune Deficiency Syndrome

AQ amodiaquine

BCG Bacillus Calmette Guerin

DHFR dihydrofolate reductase

DHPS dihydropteroate synthase

DPT diphtheria, pertussis and tetanus

DSMB date and safety monitoring board

EANMAT East-African network of monitoring malaria treatment

EIR entomological inoculation rate

EPI expanded programme of immunisation

GCP good clinical practice

Hb haemoglobin

HPLC high performance liquid chromatography

IMR infant mortality rate

IPTi intermittent preventive treatment in infants

ICH International Conference on Harmonisation

LSHTM London School of Hygiene & Tropical Medicine

MoH Ministry of Health

PCV packed cell volume

Pfmdr plasmodium falciparum multi drug resistance

SP sulphadoxine/pyremethamine

SSOP sequence specific oligonucleotide probe

VSA variant surface antigen

WHO World Health Organisation

**Summary**

Malaria and anaemia are major causes of morbidity and mortality in children in sub-Saharan Africa. Administration of three courses of sufadoxine/pyrimethamine (SP) as intermittent preventive treatment (IPTi) to infants when they receive EPI vaccines at 2,3, and 9 months of age reduced the incidence of malaria and anaemia in infants in an area with low SP resistance, low transmission pressure and high bednet use. However, it is not clear whether this observation can be generalised to areas with high transmission and high SP resistance. The mechanism of the protective effect of IPTi is unclear. The key question whether the mechanism of action is by intermittent treatment, by prophylaxis, or both remains unanswered. If it is mediated through treatment, then all effective short acting antimalarials can be used for IPTi. If, on the other hand, the predominant effect is mediated via prophylaxis, then only long acting drugs can be considered. High levels of SP resistance are recorded in Eastern and Southern Africa. SP resistance is generally lower in West Africa, but this situation will change soon as SP becomes the first line treatment in place of chloroquine which is no longer effective. Thus, SP is already unlikely to be an effective drug for IPTi in many parts of East Africa and to have only a limited lifespan in other parts of the continent. There is, therefore, an urgent need to identify other anti-malarial drugs that could be used for IPTi instead of SP.

This study has four main objectives:

1. Identification of a drug that could be used safely and effectively for IPTi instead of SP in areas, such as north eastern Tanzania, where there is a high level of resistance to SP and amodiaquine.
2. Determination of whether a short acting antimalarial drug (Lapdap) is as effective as a long acting drug (mefloquine) when used for IPTi.
3. Investigation of the effect of the intensity of transmission on the requirements for a long or short acting drug for IPTi.
4. Assessment of the effect of IPTi on the development of clinical immunity in children in low and high transmission areas.

A randomised trial with four treatment regimes is proposed which will be conducted in two different transmission settings [(Lwengera (high transmission) and South Pare (low/moderate transmission)] The four treatment regimens are as follows: (1) placebo; (2) mefloquine; (3) Lapdap; (4) SP. All medications will be given at the time of immunisation with DPT/polio 2, DPT/polio 3, and measles vaccines. The study will involve 1280 infants in Lwengera and 2440 infants in South Pare, in Tanzania.

The primary outcome measure is incidence of malaria in infancy. The secondary outcome measures include the following: (1) mean Hb at 10-12 months of age; (2) incidence of severe anaemia during infancy; (3) prevalence of malaria parasitaemia at 10-12 months of age; (4) incidence of malaria in the second year of life. (5) Level and repertoires of plasma antibodies to *Plasmodium falciparum* variant surface antigen (anti-VSA antibodies) at 10 and 18 months of age.

The time frame of the study is three years starting in July 2004. This study has no funding from any other donor agencies except the LSHTM's contribution through the time of the London based principal investigators and collaborators. The request for financial support from the Bill and Melinda Gates Foundation is $ 1 551 297.

**1. Background**

**1.1. Malaria as a cause of anaemia**

Malaria is a major cause of morbidity and mortality in children in sub-Saharan Africa. In Africa, malaria causes over a million deaths and many million episodes of illness each year in children less than five years-old. ^1^ Anaemia, caused by malaria and iron deficiency, is another leading cause of severe disease in children in Africa.^2^ Around 75% of preschool children in East Africa are anaemic ^3^ and anaemia in childhood is associated with impaired physical and mental development. ^4^ In areas of high malaria enedmicity the incidence and adverse effects of anaemia are most marked in the first 1-2 years of life. ^5^

Several studies have shown that iron supplementation increases the haemoglobin level in young children resident in malaria endemic areas.^5,6^ However, concerns have been expressed about the effect of iron supplementation on malaria.^7^ An increased incidence of malaria as a result of iron supplementation can occur under some circumstances, but there is a general consensus that the effect is not marked and is out-balanced by the beneficial effect of iron on anaemia. Daily iron supplementation given by mothers reduced the incidence of severe anaemia by 32% in infants in a highly malaria endemic area of Tanzania without increasing clinical episodes of malaria in children who received iron. ^6^ In this study, additional administration of malaria chemoprophylaxis ((weekly Deltaprim (pyrimethamine/dapsone)) led to a 57% reduction in severe anaemia and a 60% reduction in episodes of malaria. These results support iron supplementation for infants, even in malaria endemic areas, and suggest an important role for chemoprophylaxis.

Chronic parasitaemia in infants is associated with anaemia. ^8^ A study in Tanzania showed that at any point in time, the mean packed cell volume (PCV) in infants with low parasitaemia (<1000 parasite/µl) was higher than that of infants with intermediate (1000-9999 parasite/µl) or high parasite densities (≥10000 parasite/µl). This suggests that interventions which lower parasite densities in infants may reduce severe anaemia and its associated morbidity and mortality. On the other hand, it has been suggested that the life-time risk of severe malaria is influenced by an ability to develop clinical immunity early in life as a consequence of repeated infections during a period when other protective mechanisms, such as maternal antibodies and breast feeding, are operating. ^9^ Thus, it is necessary to achieve a balance in which chemoprophylaxis or intermittent treatment reduces substantially the risk of severe disease during first year of life without markedly affecting the development of immunity.

**1.2. Prevention of malaria by chemoprophylaxis or intermittent preventive treatment**

Several studies carried out in tropical Africa have shown that regular administration of antimalarial drugs throughout early childhood increases haemoglobin levels substantially. ^10,11^ However, when chemoprophylaxis is stopped there may be a rebound in the incidence of clinical malaria^12^ or severe anaemia during the following months. ^6^ Intermittent preventive treatment (IPTi) in which a full therapeutic course of antimalarials is given intermittently throughout early life provides a possible way of providing some protection against the serious clinical consequences of malaria infection without impairing the development of immunity to the infection.

In Tanzania, Schellenberg and colleagues showed that IPTi with sulfadoxine/ pyrimethamine (SP) at 2, 3, and 9 months of age given with daily iron supplementation from 2-6 months of age, reduced the incidence of severe anaemia (PCV <25%) by 50% and episodes of malaria by 59% without significant rebound infection leading to severe malaria or anaemia during the second year of life. ^13^ Another study in northern Tanzania showed that IPTi with amodiaquine (AQ) given at 3, 5, and 7 months of age plus daily iron supplementation reduced malaria fever and anaemia by 65% and 67% respectively without any significant rebound effect after discontinuing IPTi. ^14^ Although these results suggest that IPTi with SP or AQ is an efficacious and safe intervention for reducing the burden of malaria and anaemia in infants, the generalisability of these results to various ecological and transmission settings in Africa is uncertain. In the study in Ifakara, Tanzania the prevalence of parasitaemia in 12 month-old infants (4%), the incidence of clinical malaria (36/100/year) and proportion of febrile episodes attributable to malaria (14.5%) were low in the placebo group compared to the expected values in high transmission settings in Africa. The low burden of malaria in the study area in Ifakara, Tanzania is likely to be due to the fact that there was a high use of bednets in the study population (67%) and that the study infants lived within 5km from a relatively well equipped mission hospital. In contrast, the prevalence of parasitaemia in <12 month old infants in Navrongo, Ghana during the high transmission season was 60%.^15^ The proportion of febrile episodes attributable to malaria ranges from 7% to 60% in sub-Saharan Africa. ^16^ Thus, further evidence is needed before recommending the use of IPTi as a means of reducing burden of malaria in high transmission areas in rural Africa.

**1.3. The mode of action of drugs used for IPTi**

This study sets out to address a series of important, and linked, practical questions about IPTi.

The mechanism of the protective effect of IPTi is unclear. SP and AQ, the two drugs studied so far, have a long plasma half life (4 to 10 days) and their chemoprophylactic effect can last up to 4 weeks. This means that IPTi with these drugs given three times during infancy would provide chemoprophylactic protection for three months in addition to clearing parasites present at the time of their administration. If the protective effect of IPTi on the incidence of severe anaemia is primarily due to its chemoprophylactic effect, then IPTi with an antimalarial drug with a short plasma half life may not have a significant effect on the incidence of anaemia.

The question as to whether the mechanism of action is actually by intermittent treatment, by prophylaxis, or both, is not just an academic one. If it is mainly mediated by treatment, then all effective short-acting antimalarial drugs can be used, and the range of appropriate drugs is the same as that for treatment of malaria, with the expectation that local patterns of resistance can accurately predict which drugs will work for IPTi. Additionally, there are good theoretical reasons for thinking that both at the individual and at the population level, short-acting drugs are less likely to select for resistance than long-acting drugs.

If, on the other hand, the predominant effect is mediated via prophylaxis then only long-acting drugs need be considered. This simplifies decisions at one level, but may mean that IPTi policy and treatment policies diverge.

The effectiveness of short and long acting antimalarials for use in IPTi may vary according to transmission intensity. In highly endemic areas, where infants receive at least one infective mosquito bite every week (EIR >50/year), the chemoprophylactic effect of IPTi may be essential as it reduces the number of infections during infancy and thus reduces the risk of severe anaemia. However, in low endemic settings where the number of infective bites is less than one per month, the chemoprophylactic effect of IPTi may not be essential and IPTi with short acting antimalarial drugs, which results in clearance of all parasites present at the time that the drug is given, may be as effective as IPTi with long acting ones in reducing the incidence of anaemia. Studies of IPTi in areas with different, and well defined, levels of transmission are needed to clarify this point.

The effects of the duration of protection provided by a single course of treatment and the number of treatment courses given on the protective effect of IPTi on severe anaemia under different levels of transmission can be modelled using a deterministic approach (Appendix 1). The predicted reduction on incidence of severe anaemia achieved with 3 or 2 course regimes of IPTi using a long and short acting antimalarial drug in low and high transmission settings are shown in Table 1 and suggest that the effect of short acting drugs on anaemia is significantly lower than that of long acting drugs in high and low transmission areas. However, these simulations have to be validated by empirical evidence before any conclusions are reached. Resolving the issue of whether or not a long acting drug is required for effective IPTi is essential in helping to decide the potential value of other new antimalarials for this purpose as they come into clinical use. It is possible that in low transmission areas 2 courses might be adequate (Appendix 2).

Table 1. Estimated effect of IPTi using long or short acting drugs in low and high transmission areas

| **Force of Infection*** | **Average duration of chemoprophylaxis (days)** | **Number of presumptive treatments**@ | **Incidence of severe anaemia in controls^#^** | **Incidence of severe anaemia in intervention group^#^** | **Reduction in incidence** |
| --- | --- | --- | --- | --- | --- |
| Low | 28 | 3 | 102 | 71 | 30% |
| Low | 28 | 2 | 102 | 78 | 23% |
| Low | 7 | 3 | 102 | 83 | 18% |
| Low | 7 | 2 | 102 | 88 | 14% |
| High | 28 | 3 | 265 | 189 | 29% |
| High | 28 | 2 | 265 | 206 | 22% |
| High | 7 | 3 | 265 | 224 | 16% |
| High | 7 | 2 | 265 | 234 | 11% |

*Low force of infection – 35% of children infected at least once by 12 months of age

*High force of infection – 70% of children infected at least once by 12 months of age

@ 3 courses at 2,3 & 9 months of age; 2 courses at 3 & 9 months

**^#^** per 1000 person years

**1.4. Choice of drug for IPTi**

The only published study of the impact of IPTi with SP on anaemia in infants used a single dose of SP given at the ages of 2, 3 and 9 months.^13^ Two studies of IPTi in Ghana and one in Kenya, which are nearing completion, have employed SP in a similar regimen. Two new studies started recently in Mozambique and Gabon are also utilising this drug. We know of only one study in which another drug, amodiaquine (AQ), has been used for IPTi. In this trial, conducted in Tanzania in 1999-2000, AQ given on three occasions during the first year of life led to reductions in the incidence of clinical malaria and of anaemia of 65% and 67% respectively. ^14^ Thus, there is little experience of the use of drugs other than SP for IPTi. However, levels of resistance to SP are already high in parts of East and Southern Africa and are increasing rapidly in other parts of Africa. ^17^ The level of resistance to SP is particularly high in north-eastern Tanzania. A study undertaken at Muheza, Tanzania in 1999 found that 45% of children under the age of five years treated with SP were still parasitaemic 7 days after treatment. ^18^ In a further study undertaken in the same area in 2002, 42% of children treated with SP were parasitaemic on day 14 (Mutabingwa, personal communication). In southern Tanzania, a study conducted in 1999 showed a lower, but still unacceptably high level of SP resistance; 31% of children were parasitaemic 14 days after treatment.^19^ High levels of SP resistance have been recorded in many other parts of Southern and Eastern Africa. In Natal the level of resistance became so high (70%) that, in 2000, it became necessary to change from SP to artemisinin combination therapy as first line treatment of clinical malaria.^20^ The rapid spread of SP resistance across eastern Africa has been monitored during the past five years by the East African Network for Monitoring Antimalarial Treatment (EANMAT); clinical failure rates of over 25% are now found across Rwanda and Burundi. In general, resistance levels to SP are lower in West and Central Africa than in East Africa but it can be anticipated that this situation will change rapidly in the next few years as SP comes to be used more widely in West Africa where chloroquine (CQ) is no longer effective. Thus, SP is already unlikely to be a fully effective drug for IPTi in much of East Africa and to have only a limited lifespan in other parts of the continent. There is, therefore, an urgent need to identify other anti-malarial drugs that could be used for IPTi instead of SP.

Other drugs that might be used for IPTi and some of their properties are shown in Table 2. Amodiaquine (AQ), is the only one that has been used for this purpose so far.

**Table 2. Plasma half life and duration of chemoprophylactic effect of**

antimalarial drugs that might be used for IPTi

| Drug | Plasma half life  Mean (range) | Duration of chemoprophylaxis |
| --- | --- | --- |
| Sulfadoxine/  Pyrimethamine  Amodiaquine  Mefloquine  Chlorproguanil/  Dapsone  Artesunate | 7 days (4-10 days)  4 days (2-6 days)  4 days  14 days  12 hrs (6-18 hrs)  24 hrs (18-30 hrs)  6 hrs (4-11 hrs) | 4 weeks  3 weeks  4 weeks  <1 week  < 1 week |

Source of plasma half life data: Compodium of data sheets and summaries of product characteristics 1999-2000. Datapharm publications Ltd 1999.

Amodiaquine resistance is variable across Africa and does not appear to have progressed as fast as resistance to SP.^21^ In some areas, this drug could probably still be used for

IPTi but in others areas, levels of resistance to AQ are probably already too high for this . For example, in a recent study undertaken in Uganda 26% of children treated with AQ were parasitaemic on day 14 after treatment ^22,23^ and an even higher level of resistance has been recorded recently in the area of north eastern Tanzania where the proposed study will be undertaken; 32% of children were parasitaemic on day 14 after the start of treatment with AQ (Mutabingwa, personal communication). Some improvement in efficacy might be obtained by combining AQ with SP but parasitological failure at day 14 after treatment with this combination was still 25% in southern Tanzania^19^ and 18% in the proposed study area in north eastern Tanzania (Mutabingwa personal communication). In north eastern Tanzania, an alternative to SP or AQ is needed already for IPTi and this situation is likely to arise soon in other parts of Africa. Thus, we have considered alternative drugs that might be used for this purpose. We have chosen two drugs for investigation – Lapdap (chlorproguanil/dapsone), a new short acting anti-folate drug, and mefloquine a well established anti-malarial with a long half life so as to be able to investigate whether or not a long half life is an essential requirement for a drug used for IPTi. Mefloquine is a potential successor to SP if a long-acting drug is required.

Mefloquine. Mefloquine is an established anti-malarial, registered in Tanzania, which has been used widely for many years for both the treatment and the prevention of malaria. It is active against strains of *Plasmodium falciparum* that are resistant to SP, CQ or AQ and it is likely to be highly effective in north-eastern Tanzania. Mefloquine has not been adopted by public health systems in Africa because of its cost, but it is used widely in the private sector in many African countries. There have been relatively few formal trials of mefloquine in African children. In Nigeria, a 100% parasitological cure rate was found in children with clinical malaria treated with mefloquine at a dose of 25 mg/kg.^24^ However, isolates showing in vitro resistance have been detected in some areas of Africa where mefloquine had not been used previously,^25^ and some treatment failures were observed when a dose of 15 mg/kg was used to treat children with malaria in Malawi.^26^ Potential advantages of mefloquine as a drug for IPTi are the requirement for only a single dose and the fact that it provides a prolonged period of prophylaxis. The main side effects of mefloquine are vomiting and neuropsychiatric complications; the prevalence of the latter varies substantially from situation to situation but is generally believed to be in the region of 1:200 - 1000 when mefloquine is used for treatment.^27^ Most information on the side effects of mefloquine has come from studies of its use for chemoprophylaxis in travellers. In a study of 500 children under 5 years of age in Thailand, Luxemburger et al observed that the dose of mefloquine 25 mg/kg was well tolerated. They also reported that no serious toxicity and no neuropsychiatric side-effects in the study children.^28^ Mefloquine is licensed for treatment of infants above the age of two months; there are no specific contra-indications for its use in children younger than this.

Lapdap. Lapdap (chloroproguanil/dapsone) is a new anti-folate anti-malarial based on a combination of two old and widely studied drugs. Lapdap has recently been through a full clinical evaluation and it is expected that the drug will be licensed in mid 2003. Studies conducted in Kenya in the 1980s and 1990s showed that this short acting drug combination was safe and effective. Clinical trials of Lapdap have recently been conducted in 1480 children in five African countries as part of the regulatory process. In each country, Lapdap was more effective than SP and in Kenya, Gabon and Malawi the difference between groups was statistically significant.^29,30^  Detailed safety observations were made; minor side effects were recorded frequently (some of these may have been due to the underlying malaria infection). Falls in Hb in children who were glucose-6-phosphate dehydrogenase deficient were recorded more frequently in children who received Lapdap than in those who received SP but no other significant side effects were recorded. In a separate open label study conducted in infants aged 3 – 12 months the drug was again shown to be safe and effective.^29^

A number of trials of Lapdap have been conducted in the proposed study area. ^31,18^ The most important, conducted in 1989/99, showed that 41/43 children who had failed treatment with SP responded fully to treatment with Lapdap.^18^ Thus, Lapdap is likely to be effective when used for IPTi in this area of Africa where levels of SP and AQ resistance are high.

Advantages of Lapdap as a potential drug for use in IPTi are that it is likely to be cheap and that its short action will diminish the chances of inducing resistance; its disadvantage is that a full therapeutic course requires drug administration over a period of three days.

There is a strong move towards the use of combination therapy for the treatment of clinical malaria, in particular combinations involving artemisinins.^32^ Whether this should also be the case for IPTi is currently uncertain but a case can be made for establishing the efficacy of alternative drugs to SP for IPTi initially as monotherapy before considering the option of combining them with artesunate prior to more widespread implementation. Mefloquine has already been used widely in combination with artesunate in South Eastern Asia and shown to be effective and safe and the use of this combination may have slowed down the rate of development of mefloquine resistance. Thus introduction of mefloquine/artesunate for IPTi would be unlikely to raise any major safety issues if mefloquine was found to be effective and safe. Lapdap has not yet been studied in combination with artmesinins and so this combination could not be used for IPTi at this time. However, a fixed combination of Lapdap and artesunate (CDA) is being developed and this might prove to be a suitable drug for IPTi if it can be shown that short-acting drugs are as effective as long acting drugs for this purpose.

**1.5. Potential effect of IPTi on clinical immunity**

A potential negative influence of IPTi on clinical immunity to malaria, which is normally acquired naturally as the consequence of repeated *P. falciparum* infections, remains an import question. This might lead to a rebound effect on malarial morbidity with an increased incidence of mild or severe malaria in the second year of life, despite the beneficial effect of IPTi on the burden of disease during the first 12 months. Protective immunity has been shown to be mediated, at least partly, by the acquisition of agglutinating antibodies against parasite-encoded, variant-specific antigens on the surface of infected red blood cells, the variant surface antigens.^33,34^ Protection is related to the acquisition of a broad repertoire of anti-VSA antibodies, and antibodies against a specific subset of parasite antigens that are particularly well expressed during infections in young children have been shown to be related to protection from severe disease during the first few years of life.^35-37^ Studies have further shown that asymptomatic low-density parasitaemia in children in itself is linked to a lower incidence of uncomplicated malaria.^38^ Together, these findings point to the importance of asymptomatic,low-grade parasitaemia in the induction of protective anti-VSA antibodies during infancy, particularly against severe forms of disease; a protection that may be disrupted by a repeated clearance of parasitaemia by presumptive intermittent treatment. Thus, it is important to assess the interaction between IPTi and the development of clinical immunity in children.

**1.6 The influence of G6PD deficiency and human haemoglobinopathies**

Glucose-6-phosphate (G6PD) deficiency and human haemoglobinopathies, such as sickle cell trait and alpha-thalasseamia are (important) inherited genetic factors, which may influence the outcome of IPTi^43,44^. G6PD-deficient red blood cells are more susceptible to oxidative stress, and haemolytic anaemia more prone to develop in G6PD-deficient patients receiving antimalarial drugs such as SP and Lapdap^45^. By monitoring the prevalence of G6PD deficiency in the study population, it will be possible to predict the risk of haemolytic anaemia among the study participants treated with these drugs, and further to adjust for the effect of G6PD deficiency when analysing the effect of different IPTi drugs on the prevalence of anaemia. Furthermore, by testing for these genetic factors, which have been associated with innate resistance to malaria, it will be possible to adjust for their influence when assessing the possible influence of different IPTi drugs on malaria morbidity and acquired immunity.

1. **Objectives**

**2.1 Co-primary objectives**

This study has four main objectives

- Identification of a drug that could be used safely and effectively for IPTi instead of SP in areas, such as north eastern Tanzania, where there are high levels of resistance to SP and amodiaquine.
- Determination of whether a short acting antimalarial drug (Lapdap) is as effective as a long acting drug (mefloquine) when used for IPTi.
- Investigation of the effect of the intensity of transmission on the requirements for a long or short acting drug for IPTi.
- Assessment of the effect of IPTi on the development of clinical immunity in children in low and high transmission areas.

2.2 Secondary objective

- To determine whether IPTi through the EPI interacts with the immune response to routine immunisations
- Assessment of the effect of G6PD deficiency and haemoglobinopathies on the outcome of IPTi.

**3. Study area**

The study will be conducted in two sites in Tanzania: (1) a low/moderate transmission area in South Pare district ; (2) a high transmission area in western Usambara district A

**Figure1: Parasite prevalence in the Kilimanjaro region**


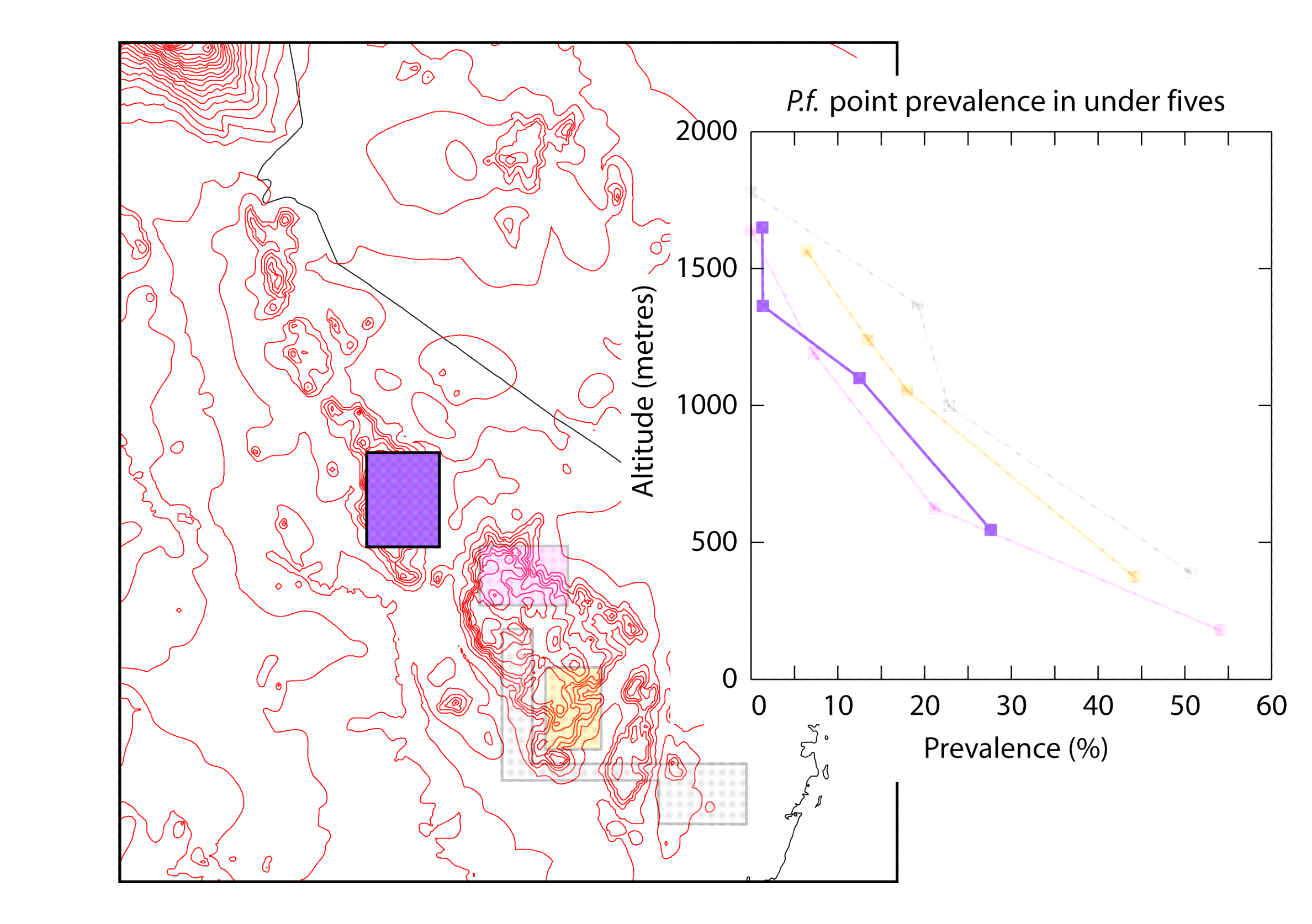


Source: Drakeley C, Reyburn et al. MIM conference abstract no. 318, Arusha, Tanzania

malariometric survey in 2002 showed significant variation in the prevalence of malaria parasitaemia in <5 year old children at different levels of altitudes within and between the

districts (Figure 2). The survey was conducted in four groups of villages: Rombo (purple), South Pare (pink), Western Usamabara (yellow) and Eastern Usambara (grey). The relationship between the mean altitude of the village and the prevalence of parasitaemia is shown in the graph (insert). The mean altitude of villages within in each group ranged from 200 to 1800 meters. The prevalence of malaria parasite in 0-4 year old children ranged from 20% to 55% in the lowlands (<750m) and from 1% to 20% at 1000-1500m altitude. The malaria transmission pressure in lowland villages in the South Pare district is low/moderate. The prevalence of malaria parasitaemia was 30% in 1-4 year-old children and 4% in <1 year-old infants. The prevalence of parasitaemia was higher in Western Usambara villages (52% in 1-4 year-old children and 20% in <1 year-old infants).

The advantages of the study site for the proposed trial are:-

1. Levels of resistance to SP and amodiaquine are high and so investigation of alternative drugs that could be used for IPTi is needed urgently and can be justified ethically.
2. A wide range of levels of malaria transmission are found over a small area in a homogeneous population.
3. The epidemiology of malaria in the study area has recently been carefully defined.

Although detailed epidemiological studies have recently been undertaken in the study area, it will be necessary to confirm that major differences persist between highland and lowland sites during the period of the trial. We have considered the possibility of making direct measurements of EIR but this is difficult and expensive in low transmission areas because of the paucity of anopheline mosquitoes and the low sporozoite rate. In a recent study in Hai district, Tanzania, 2198 anopheline mosquitoes were collected over a period of 12 months and only 8 mosquito were infected giving an EIR with very wide confidence limits. Instead of EIR estimates, we will rely upon the prevalence of infection in nine month old infants in the placebo groups to estimate the force of infection in the two sites.

The number of infants receiving DPT1 (the expected number of infants that can be enrolled in a year) and laboratory facilities available in the health facilities serving the populations of South Pare and Western Usambara are shown in Tables 3 and 4

**Table3. Infant population and infrastructure of health facilities in South Pare**

| Name of health facility | Number of infants receiving DPT1 in 2002 | OPD cases in children <5 years-old in 2002 -  total | OPD cases in children <5 years-old in 2002 -  malaria | Laboratory facility | Power supply |
| --- | --- | --- | --- | --- | --- |
| Ndungo | 437 | 4025 | 1256 | Microscopy | electricity |
| Kisuwani | 273 | 3284 | 1291 | None | Kerosene |
| Kiturio | 196 | 5212 | 2135 | Microscope | Electricity |
| Bandera | 58 | 1821 | 556 | Microscope | Electricity |
| Gonja | 230 | 2615 | 1086 | Microscope | Electricity |
| Mpirani | 122 | 314 | 183 | Microscope | Electricity |
| Same | 638 | NA | NA | Microscope | Electricity |
| Mbono | 97 | 1030 | 384 | None | Kerosene |
| Vutima | 151 | 100 | 36 | None | Kerosene |
| Vumari | 72 | 1232 | 408 | None | Kerosene |

**Table4: Infant population and infrastructure of health facilities in Western Usambara**

| Name of health facility | Number of infants receiving DPT1 in 2002 | OPD cases in children <5 years-old in 2002 -  total | OPD cases in children <5 years-old in 2002 -  malaria | Laboratory facility | Power supply |
| --- | --- | --- | --- | --- | --- |
| Mbaramo | 312 | 3036 | 1047 | None | Kerosene |
| Mlalo | 601 | 8268 | 5877 | Microscope | Electricity |
| Kangagai | 141 | 1692 | 828 | Microscope | Electricity |
| Ngwelo | 260 | 1671 | 684 | None | Kerosene |
| Mnazi | 381 | 3646 | 1773 | Microscope | Kerosene |
| Kivingo | 94 | 1562 | 1022 | None | Kerosene |
| Mng’aro | 185 | 1755 | 1527 | None | Kerosene |

respectively. The study will be conducted in five health facilities in the South Pare district (Ndungo, Kisiwani, Gonja, Mpirani, Same) and in five in Western Usambara district (Mbaramo, Mlalo, Kangagai, Ngwelo, Mnazi). These health facilities were selected on the basis of the number of infants needed for the study (see below) and logistical feasibility.

**3.1. Change of study site**

A site visit by the principal investigators (Daniel Chandramohan, Martha Lemgne, Frank Mosha) to the proposed study site in June 2004 showed that two health facilities (Mlalo and Ngwelo) and their catchment areas are situated at an altitude >800 meters and that malaria transmission would be medium/low in these two populations. Hence infants attending these two health facilities, cannot be included in this study because Western Usambara is the high malaria transmission site chosen to address the question on differences in the efficacy of IPTi between high and medium/low transmission areas. Restricting the enrolment of infants to the remaining three health facilities in the Western Usambara will reduce the number of eligible study infants substantially and it will not be possible to enrol the required number of infants (1280) in 12 to 15 months. Therefore, another high transmission site is needed.

The transmission of malaria in the Lwengera valley in Korogwe district is high. The prevalence of malaria parasitaemia in <5 year old children in this area was 50-60% during the baseline survey conducted in 2002. The PIs met the Korogwe District Medical Officer (DMO) and his colleagues and discussed the possibility of conducting the IPTi study in Lwengera valley. The DMO and his team are very cooperative and supportive of this move. The PIs visited the health facilities and the surrounding villages in Lwengera valley in Korgowe district on the 7^th^ of June 2004 and assessed the appropriateness and feasibility of conducting the study in Lwengera valley. The health facilities serving the population of Lwengera valley and the number of infants that could be recruited for this study in a year are shown in Table 5.

As a result of those discussions, a request to move the study from Lushoto to Korogowe district has been submitted to the ethics committees in Tanzania and at the London School of Hygiene & Tropical Medicine.

Table 5. Number of infants receiving DPT2 in Lwengera valley health facilities, 2003

| Health Facilities | Number of infants that received DPT2 in 2003 |
| --- | --- |
| Korogwe hospital MCH  Majongo dispensary  St Raphael Hosptial  Lwengera dispensary  Magazini dispensary  Mnyuzi dispensary | 250  200  50  160  140  180 |
| Total | 980 |

**3.2 Addition of Hale Dispensary as study site in Lwengera Valley**

Hale Dispensary has not been included in the main IPTi study and is being used as the study site for the IPTi Efficacy studies (NIMR ref: NIMR/HQ/R.8a/Vol. IX/434).

**4. Study Methodology**

**4.1. Study design**

The overall study design is a randomised trial of four treatment regimes in two different transmission settings. The key steps and the arms of the study are shown in Figure 2. The four treatment regimes are as follows: (1) placebo, (2) mefloquine; (3) Lapdap; (4) SP. All medications will be given at the time of immunisation with DPT/polio 2, DPT/polio 3, and measles vaccines.

**4.2. Study population : inclusion/exclusion criteria**

The study population will be all infants attending EPI clinics at the 10 study health facilities (listed in section 3) for first vaccinations. Infants who live within the catchment area of the study health facility and are less than 3 months of age at the time of DPT and Polio 1 vaccination will be eligible for inclusion in the study. Infants having any of the following conditions will be excluded: (1) history of allergy to study drugs; (2) history of convulsions; (3) clinical features of severe malnutrition or chronic illness including infants with signs of AIDS [HIV prevalence in women of reproductive age in the study area was 11.5% in 1999]^39^ (4) plans to leave the study area before 12 months of age.(5) weighs <4.5 kgs (6) caretaker declines to give consent.

To assess how representative the sample population is of the population as a whole we will carry out a demographic survey of the study sites during the time of enrolment. This survey will take a random sample of dwellings within the study area and numerate the number of children under 5 years with an MCH/Vaccination card, numerate those eligible for enrolment into the study by age and those that have been enrolled into the study. Using census data we will extrapolate our results.

**Figure 2**. **Study design**

**Contact 0**: Infants attend EPI clinics for BCG and/or DPT & Polio1 vaccines

1. identification of eligible infants

2. provision of information about the study to the care takers

3. enrolment of eligible infants after obtaining informed consent

2 months

Approximate age

**Contact 1:** Infants return for DPT 2 and Polio 2 vaccines

1. random allocation to one of the study groups

2. administration of the first course of treatment

3. collection of blood samples for Hb and blood film and filter paper

4. appointment for contact 2 (DPT and polio 3)

Group 1

placebo

Group 2:

Mefloquine

Group 3:

Lapdap

Group 4:

SP

Passive surveillance at health facilities for side effects of drugs, clinical malaria, and anaemia until 24 months of age

**Contact 2**: Infants attend EPI clinic for DPT 3 & Polio 3 vaccines

1. completion of data forms and administration of the second course of allocated drug

2. appointment for contact 3 (measles vaccine)

**Contact 3**: Infants attend EPI clinic for measles vaccine

1. completion of data forms and administration of the third course of allocated drug
2. collection of blood samples for baseline measles serology
3. appointment for contact 4 (1 month after the date of contact 3)

**Contact 4**: (1 month after the date of contact 3)

Collection of blood samples for Hb, blood film, post vaccine measles serology, and anti-VSA antibody assay

1 months

3 months

9 months

10 months

**Contact 5**: (20% random sample)

Collection of blood sample for Hb and blood film

**Contact 6**: (20% random sample)

Collection of blood sample for Hb and blood film

**Contact 7**:

Collection of blood sample for Hb, blood film & antibody assay

11 months

12 months

18 months

**Contact 8:**

for Hb, blood film, & filter paper, and screening by Paracheck dipsticks for malaria parasites

Random allocation of treatment for malaria for those +ve for malaria parasites by dipstick

**Contact 9: (Blood film +ve at contact 8 only)**

Collection of blood sample for blood film and filter paper

**Contact 10: (Blood film +ve on contact 8 only)**

Collection of blood sample for blood film and filter paper

24 months

Post treat-ment day 14

Post treat-ment day 28

**4.3. Outcome measures**

*Primary outcome measures*

1. Incidence of clinical malaria: [(history of fever during previous 2 days or axillary temperature >37.5ºC) + parasitaemia of any density + absence of any other obvious causes of fever] during the period of 2-11 months of age

*Secondary outcome measure*

1. Mean Hb at 10-12 months of age (one month after the third course of IPTi)
2. Incidence of severe anaemia (Hb <7 g/dl) during the period of 2-11 months of age
3. Prevalence of parasitaemia at 10-12 months of age (one month after the third course of IPTi)
4. Incidence of clinical malaria [as defined above] during the period of 12-23 months of age.
5. Level and repertoires of plasma antibodies to *plasmodium falciparum* variant surface antigen (anti-VSA antibodies) at 10 and 18 months of age.
6. EPI serology
7. Prevalence of G6PD deficiency and human haemoglobinopathies.

**4.4. Sample size**

The number of infants per arm needed for a study with 80% power and 95% significance for the primary and secondary outcome measures in the low/moderate transmission site

(South Pare) is shown in Table 6a. The required sample size was calculated using STATA software and based on the following assumptions:

1. the incidence of clinical malaria and anaemia in the placebo group in this setting will be similar to that observed by Schellenberg et al in Ifakara, Tanzania in 1999-2000;^13^
2. The effect of IPTi with mefloquine on clinical malaria and anaemia is similar to that of IPTi with SP observed by Schellenberg et al.;^13^
3. The effect of IPTi with Lapdap on clinical malaria and anaemia will be 50% less than the effect of mefloquine because Lapdap has little or no chemoprophylactic effect;
4. The effect of IPTi with SP on clinical malaria and anaemia will be 50% less than the effect of mefloquine because the resistance to SP in this setting is expected to be 50%.

Low transmission site: For the comparison of the primary outcome measure, the highest number infants (551) needed is for the comparison of the incidence of malaria between placebo and Lapdap groups. Allowing for 10% loss to follow up, 610 infants per arm i.e. a total of 2440 infants would be needed for the study in the South Pare district (see table 6a).

This sample size will have the power (90%) to detect a difference of 0.5g/dl Hb or less between the mefloquine and Lapdap arms, and also to detect a difference of 0.5g/dl between all treatment arms and placebo.

**Table 6a:** Estimates of the incidence or prevalence of primary and secondary outcomes in the comparison groups and the sample sizes required for a study in South Para area (low/moderate transmission)

| Endpoints | Expected rate/prevalence | | | Sample size per arm needed for a study with 80% power and 95% significance | | |
| --- | --- | --- | --- | --- | --- | --- |
|  | Placebo group | MQ group | Lapdap and SP groups | Placebo versus  MQ group | Placebo versus Lapdap or SP group | MQ versus  Lapdap or SP group |
| **Primary**   1. Incidence of clinical malaria in infants   **Secondary**   1. Mean Hb at 10 months of age 2. Incidence of severe anaemia in infants 3. Prevalence of parasite at 10 months of age 4. Risk of clinical malaria at 12-23 months | 0.40/child/yr^a^  9.5 gm/dl^c^  0.16/child/yr^a^  30%^c^  0.40/child^f^ | 0.20/child/yr^a^  10.5 gm/dl^d^  0.08/child/yr^a^  15%^e^  0.56/child^g^ | 0.30/child/yr^b^  10.0 gm/dl^b^  0.12/child/yr^b^  20% ^e^  0.48/child^h^ | 118  63  295  121  295 | **551**  252  1,377  294  628 | 393  252  984  908  636 |

^a^Based on estimates from Ifakara in 1999-2000 (Schellenberg et al) in the placebo and SP groups.

^b^Assumes that the effect of Lapdap on clinical malaria and anaemia will be 50% less than MQ because Lapdap has very little or no chemoprophylactic effect and that the effect of SP will also be 50% less than MQ because the resistance to SP in this area is 50%.

^c^Based on estimates from surveys done in Kilimanjro region in 2001-2002 (MRC-1 and Hai projects - unpublished).

^d^50% reduction in the incidence of severe anaemia was based on the observation in Ifakara in 1999 and it is assumed that this would increase the mean Hb at 10 months of age by 1 gm/dl.

^e^ Assumes that the reduction in the prevalence of parasitaemia at 10 months of age would be similar to the reduction in the incidence of clinical malaria.

^f^Assumes that the incidence of malaria in the second year of life would be similar to the first year and that 25% of these infection will be severe.

^g^Assumes that incidence of malaria in the MQ group will increase by 40% in the second year of life

^h^Assumes that the incidence of malaria in the Lapdap and SP groups will increase by 20% in the second year of life.

The number of infants per arm needed for a study with 80% power and 95% significance for the primary and secondary outcome measures in the high transmission site (Western Usambara) is shown in Table 6b. For the comparison of the primary outcome measures, the highest number infants (291) needed was for the comparison of incidence of malaria between placebo and Lapdap groups. Allowing for 10% loss to follow up, 320 infants per arm ie. a total of 1280 infants would be needed for the study in the Western Usambara district.

For the comparison of the incidence of clinical malaria in the second year of life (rebound effect) the data from the two sites has to be combined. This combined population would have adequate power to compare the incidence between the placebo group and the mefloquine group. However, this sample size may not be adequate for comparing between the placebo and Lapdap group unless the rebound effect is as high as that in the mefloquine group.

**Table 6b:** Estimates of the incidence or prevalence of primary and secondary outcomes in the comparison groups and the sample sizes required for a study in Western Usambara area (high transmission)

| Endpoints | Expected rate/prevalence | | | Sample size per arm needed for a study with 80% power and 95% significance | | |
| --- | --- | --- | --- | --- | --- | --- |
|  | Placebo group | MQ group | Lapdap and SP groups | Placebo versus  MQ group | Placebo versus  Lapdap or SP group | MQ versus  Lapdap or SP group |
| **Primary**   1. Incidence of clinical malaria in infants   **Secondary**   1. Mean Hb at 10 months of age 2. Incidence of severe anaemia in infants 3. Prevalence of parasite at 10 months of age 4. risk of clinical malaria at 12-23 months | 0.62/child/yr^a^  9 gm/dl^c^  0.39/child/yr^a^  40%^c^  0.62/child^f^ | 0.30/child/yr^a^  10 gm/dl ^d^  0.20/child/yr^a^  20% ^e^  0.86/child^g^ | 0.45/child/yr^b^  9.5 gm/dl^b^  0.30/child/yr^b^  30% ^e^  0.74/child^h^ | 71  63  129  81  59 | **291**  252  670  357  252 | 262  252  393  294  190 |

^a^Based on estimates from Ifakara in 1995 (Menendez et al) in placebo and Deltaprim groups

^b^Assumes that the effect of Lapdap on clinical malaria and anaemia will be50% less than MQ because Lapdap has very little or no chemoprophylactic effect and that the effect of SP will also be 50% less than MQ because the resistance to SP in this area is 50%.

^c^Based on estimates from surveys done in Kilimanjro region in 2001-2002 (MRC-1; unpublished).

^d^50% reduction in the incidence of severe anaemia was based on the observation in Ifakara in 1999 and it is assumed that this would increase the mean Hb at 10 months of age by about 1 gm/dl.

^e^ Assumes that the reduction in the prevalence of parasitaemia at 10 months of age would be similar to the reduction in the incidence of clinical malaria.

^f^Assumes that the incidence of malaria in the second year of life would be similar to the first year and that 20% of these infection will be severe.

^g^Assumes that incidence of malaria in the MQ group will increase by 40% in the second year of life

^h^Assumes that the incidence of malaria in the Lapdap and SP groups will increase by 20% in the second year of life.

**4.5. Screening, enrolment and follow up procedures**

All infants attending the EPI clinics at the study health centres for BCG or DPT&polio 1 vaccination (**contact 0**) will be screened for eligibility for enrolment into the study. The study objectives and procedures will be explained to caretakers of the infants who are eligible and they will be given an information sheet to take home. After obtaining written consent, the following procedures will be carried out: (1) collection of digital photos for a photo ID card; (2) completion of an enrolment form which will contain information on identification, address and other details useful for follow up at home, socio-economic status, use of bednets and other malaria prevention measures, clinical history and signs and anthropometric indicators.

A list of study infants due for DPT/Polio 2 vaccination in each study EPI clinic on a given date will be generated from the enrolment database a week prior to the scheduled date. A member of the project team will visit the homes of these infants and remind them to attend the scheduled EPI clinic.

When the study infants return for DPT& Polio 2 (**contact 1**) the following procedures will be carried out: (1) allocation of infants to one the four arms of the study; (2) administration of the first dose of the study drug and dispensation of the remaining two doses of drug to be given at home; (3) collection of blood samples for Hb, malaria parasite and blood spot for genetic markers of drug resistance parasites; (4) advice given to the caretakers to bring their children to the health facility if the child becomes ill before the next scheduled visit.

In both study areas, the first 200 children will be followed up on day 7 post-administration of IPTi first course (**contact 1a**) and a finger prick blood sample will be collected for determination of haemoglobin and preparation of blood film.

A list of study infants due for DPT/Polio 3 vaccination in each study EPI clinic on a given date will be generated from the contact 1 database a week prior to the scheduled date. A member of the project team will visit the homes of these infants and remind them to attend the scheduled EPI clinic.

When the study infants return for DPT& Polio 3 (**contact 2**) the following procedures will be carried out: (1) administration of the first dose of the second course of the study drugs and dispensation of the remaining two doses of drugs to be given at home; (2) advice given to the caretakers to bring their children to the health facility if the child becomes ill before the next scheduled visit.

A list of study infants due for measles vaccination in each study EPI clinic on a given date will be generated from the contact 2 database a week prior to the scheduled date. A member of the project team will visit the homes of these infants and remind them to attend the scheduled EPI clinic.

When the study infants return for measles (**contact 3**) the following procedures will be carried out: (1) administration of the first dose of the third course of the study drugs and dispensation of the remaining two doses of drugs to be given at home; (2) collection of blood samples for measles serology; (3) advice given to the caretakers to bring their children to the health facility if the child becomes ill.

In both study areas, the second 200 children will be followed up on day 7 post-administration of IPTi third course (**contact 3a**) and a finger prick blood sample will be collected for determination of haemoglobin and preparation of blood film.

A list of study infants who are due for blood examination one month after receiving course 3 of the study drugs will be generated from the contact 3 database every month. These infants will be invited to attend the clinic where they will be reviewed and **(contact 4)** a finger prick blood sample (400 μl) will be collected for determination of Hb, blood film preparation for malaria parasites and measuring anti-VSA antibodies.

A random sample of 20% of infants will be invited to attend the clinic at 11 months of age or two months after the 3^rd^ course of the study drug (**contact 5**) and a finger prick blood sample will be collected for Hb and blood film preparation.

Another random sample of 20% of children will be invited to attend the clinic at 12 months of age or 3 months after the 3^rd^ course of the drug (**contact 6**) and a finger prick blood will be collected for Hb and blood film preparation. Infant who had a blood sample taken at 11 months of age will be excluded from the sample of infants selected for collection of blood samples at 12 months of age.

All study children will be invited to attend the clinic at the age of approximately 18 months (**contact 7**) and a finger prick blood sample (400 μl) will be obtained for determination of Hb, blood film preparation for malaria parasites and measuring anti-VSA antibodies.

# Change in Contact 7 for Low transmission site

Contact 7 at age 18 months will be the last scheduled visit for children at the low transmission site. This is because the low incidence in malaria at the site means that there is a very small chance that this study will detect a rebound effect of malaria even if present.

At the high transmission site, all children will be invited to attend the clinic at the age of approximately 24 months (**contact 8**) and finger prick blood sample will be obtained for screening for malaria using Paracheck dipsticks and also for preparation of blood slides, filter paper samples and Hb. If a child is positive dipstick test is currently febrile (Temp >37.5° C) or has a history of fever with in the past 2 days, and/or has a history of taking SP for a febrile illness during the previous week, the child will be treated with Artemether - Lumefantrine.

In Korogwe District, the high transmission site the following study will take place. If the dipstick test is positive for malaria and there is no history of fever within the past 2 days and no history of use of antimalarials within the past week, the child will be given a full treatment course of any one of the three antimalarial drug used for IPTi ie SP, Lapdap or mefloquine, allocated randomly. The randomisation of treatment regime will be done in blocks of 6. The study will be open label. Within each block of 8 envelopes, four will contain Lapdap for 3 days and four will contain MQ. SP will not be tested due its low efficacy. The day 1 drug will be given under supervision. The drugs for day 2 and day 3 will be handed to the caretaker who will be given instructions about the timing of the day 2 and day 3 doses. Field workers will visit all randomised children at home on days 2, 3 and 4 to assess symptoms and measure the child’s temperature. The field workers will check that the Lapdap dose days 2 and 3 have been correctly given. All children who were treated with an antimalarial drug will be followed up on post treatment day 7, 14 (**contact 8a & 9),** 21 and on day 28 **(contact 9a & 10).** To ascertain the preventive effect of the study drugs, we will add in 2 additional visits on days 42 and 56 (contacts 11 and 12). On these two visits a finger prick blood sample will be taken for examination of malaria parasites and filter paper sample obtained for subsequent determination of resistance markers to SP and Lapdap. Children with fever (temperature greater than 37.5 ° C) at any time in the follow up will have a blood slide, filter paper sample and haemoglobin measurement. They will receive rescue treatment of Artemether- Lumefantrine if they fulfil the Standard WHO efficacy treatment failure categories of; Day 3 parasitaemia greater than Day 1, Day 4 parasitaemia greater than 25% of Day 1, any parasitaemia after Day 4 or they develop of any sign suggesting severe malaria.

**4.6. Randomisation and allocation of study groups**

The unit of randomization will be an individual infant. Infants will be allocated to one of the study groups by permuted block randomization. Each study drug, including placebo, will be assigned 4 group codes; there will be 16 study groups in total in each study site and these will be colour coded. An independent statistician (Dr Tom Smith) will prepare the randomization scheme during the preparatory phase of the study. 233 blocks of 16 blister packs of study drugs [80 blocks (1280/16 = 80) for the high transmission area, and 153 blocks (2448/16 = 153) for the low transmission area] will be prepared and the study drug codes will be kept by the independent statistician.

- 1. **Dose schedule and administration of study drugs**

The dose schedules of the study drugs are shown in Table 7a and 7b and the drug package schemes are shown in Appendix 4 and 5.

**Table 7a. Dose schedule of study drugs for IPTi course 1 & 2**

| **Study group** | **Dose on day 1** | **Dose on day2** | **Dose on day3** |
| --- | --- | --- | --- |
| Placebo | 1 tab CD placebo^$^  ½ tab SP placebo | 1 tab CD placebo | 1 tab CD placebo |
| SP | 1 tab CD placebo  ½ tab SP^£^ | 1 tab CD placebo | 1 tab CD placebo |
| Mefloquine | 1 tab CD placebo  ½ tab Mefloquine@ | 1 tab CD placebo | 1 tab CD placebo |
| Lapdap (CD) | 1 tab Lapdap^#^  ½ tab SP placebo | 1 tab Lapdap | 1 tab Lapdap |

^$^ tablets of lactose and maize starch, looking similar in size, shape and colour to Lapdap

^£^ sufadoxine 500mg + pyrimethamine 25mg per tab

@ mefloquine 250mg per tab

^#^ Chlorproguanil 15mg + dapsone 18.75mg per tab (CD/Lapdap)

**Table 7b. Dose schedule of study drugs for IPTi course 3**

| **Study group** | **Dose on day 1** | **Dose on day2** | **Dose on day3** |
| --- | --- | --- | --- |
| Placebo | 1 ½ tab CD placebo^$^  1 tab SP placebo | 1 ½ tab CD placebo | 1 ½ tab CD placebo |
| SP | 1 ½ tab CD placebo  1 tab SP^£^ | 1 ½ tab CD placebo | 1 ½ tab CD placebo |
| Mefloquine | 1 ½ tab CD placebo  1 tab Mefloquine@ | 1 ½ tab CD placebo | 1 ½ tab CD placebo |
| Lapdap (CD) | 1 ½ tab Lapdap^#^  1 tab SP placebo | 1 ½ tab Lapdap | 1 ½ tab Lapdap |

^$^ tablets of lactose and maize starch, looking similar in size, shape and colour to Lapdap

^£^ sufadoxine 500mg + pyrimethamine 25mg per tab

@ mefloquine 250mg per tab

^#^ Chlorproguanil 15mg + dapsone 18.75mg per tab (CD/Lapdap)

The mean age of study infants when they receive the third course of IPTi would be 9 months and the average body weight of children at 9 months of age is 8.5 kgs for girls and 9.5 kgs for boys. Thus, it is necessary to increase the dose of antimalarial drugs for the third course of IPTI. The dose of SP and MQ will be increased to 1 tab and that of Lapdap will be increased to 1 ½ tablets per day for three days.

The tablets will be crushed and mixed with honey at the time of administration. The first dose will be administered at the EPI clinic by a designated member of the study team.

Children will be observed for 30 minutes, and a repeated dose will be given if vomiting occurs within this time period.

The drugs for day 2 and 3 will be dispensed in blister packs and the caretakers will be advised to administer the drugs at home. The level of compliance and side effects of the drug will be monitored by the compliance assessment team during home visits made on day 2 and day 3. If drugs had not been given by the caretakers, the project staff will administer the drugs with the consent of the caretaker.

Oral administration of mefloquine can induce vomiting in children suffering from clinical malaria. Administration of mefloquine in two divided doses over two days may reduce the risk of vomiting. However, a single dose regime is preferable for directly observed administration of the drug in routine programme conditions. As most of the infants attending EPI clinics will be asymptomatic, the incidence of mefloquine induced vomiting may be low. We will observe the incidence of vomiting over an initial period of three months and report this information to the Data, Safety and Monitoring Board (DSMB). If the incidence of vomiting is significant, the DSMB may recommend that the schedule of mefloquine administration should be changed to a split dose (15 mg /kg on day 1 and 10 mg/kg on day 2).

It has not been feasible to obtain identical looking placebos for mefloquine, we will use placebo tablets for Lapdap and SP only. All children will receive the same number of tablets. Staff who administer the initial course of treatment at the EPI clinics may not be totally blind to the study groups but they will not play no further part in the trial. Mothers administering further treatment at home, and the field staff who supervise this will be blinded as to whether the child is receiving Lapdap or placebo. All field staff involved in making observations on side effects and morbidity and all laboratory staff will be blind to the nature of an infant's study group.

- 1. **Monitoring compliance and safety**

Compliance with the three days of medication will be assessed by field staff during their visits on days 2 and 3. To confirm that a high level of accuracy is achieved in administration of the correct drug, approximately 336 finger heel prick filter paper samples (112 for each drug group) will be collected at about the mid-point of enrolment on day 1 and day 3 and tested for mefloquine, dapsone and pyrimethamine using high performance liquid chromatography (HPLC).

Determining the safety and acceptability of mefloquine and Lapdap when used for IPTi will be a major objective of the study. This will be accomplished in three main ways:

(1) Changes in haemoglobin during the week following treatment will be measured in the first 400 children (200 after first course of IPTi and the second 200 after the third course of IPTi) and related to the presence or absence of parasitaemia and or fever at the time of treatment. Data for each of the 16 treatment groups will be collated by the investigators and given to the DSMB which will be asked to break the code for these subjects and to determine if there is are any concerns over a fall in Hb in the approximately 200 infants who have received Lapdap. It will be possible to compare findings in these children with those children who receive mefloquine, SP or placebo. If necessary, the G-6-P-D status will be determined in any children who show a drop in Hb and in matched controls. Based on the results of these findings the DSMB will be asked to make recommendations as to whether collection of any additional safety data is required.

(2). At the time of the day 3 follow-up, a questionnaire will be completed which asks about the health of the infant since the time of drug administration; particular attention will be paid to the occurrence of vomiting.

(3). An adverse events reporting system will be established in each of the health centres participating in the trial. Any illness that occurs within 28 days of administration of a study drug will be documented on an adverse events form . Any severe adverse event, as defined in appendix 2, will be reported to the Dr Watkins, the chair of the DSMB within 1 week of the report being received. In addition, all serious adverse events will be reported to GSK regional office (fax no. + 254 020 558329/534204) with in 1 week of becoming aware of the event using the SAE form provided by GSK. A copy of the drug codes will be provided to Dr Watkins.

The study is not powered to detect differences between groups in the incidence of severe malaria. However, records will be kept of all study children admitted to Same or Korogwe hospitals with severe malaria or who require blood transfusion for anaemia.

Infant mortality in the study area remains quite high (IMR 90 per 1,000 live births) so at least 100 deaths can be anticipated in study children. Mortality will not be a trial end-point, as the sample size is not sufficient for this but, on safety grounds, all deaths will be investigated by the post-mortem questionnaire technique and reported to the DSMB.

Drugs used for IPTi must not have any suppressive effect on the immune response to EPI vaccines given concurrently. Investigation of possible interactions of this kind is being co-ordinated by a committee set up for this purpose by WHO. The possible impact of SP is being studied in a number of other IPTi with SP trials and will not be investigated in this trial. However, no other study being conducted under the auspices of the IPTi consortium is investigating the effect of mefloquine. An impact on measles vaccination is the issue of most concern. Therefore, a serum sample will be collected at the age of 9 months before administering measles vaccination and one month after the administration of the vaccine. This will also be tested for the impact of Lapdap on measles vaccination. Antibody concentrations to DPT antigens will also be measured in these samples.

- 1. **Surveillance of clinical malaria**

All study infants will be encouraged to attend the study health facilities for any illness. At these visits a health questionnaire will be completed and temperature recorded using an electronic thermometer. A finger prick blood sample will be collected for Hb and malaria parasite and for detection of molecular markers of resistance from all infants attending the study health facilities for any illness that is clinically diagnosed as malaria. At the health centres one blood slides will be examined on the same day for deciding on appropriate treatment whilst the second slide will be read in the central laboratory to provide definitive diagnosis.

Children with proven or presumptive malaria will be treated according to the MoH guidelines for managing treatment failures following first line drugs. We have opted for the second line antimalarials instead of giving SP, the first line drug recommended by the MoH, because we believe that most of the break through infections will be in the SP and placebo groups and therefore a repeat dose of SP will not be appropriate in this setting where there is a high level of SP resistance.

To ensure that IPTi with any of the study drugs does not lead to a rebound in the incidence of severe malaria or anaemia during the second year of life all infants enrolled in the study will be followed until they reach the age of 24 months. During their second year of life, all episodes of possible malaria or anaemia in infants who present to health centre in the study area will be documented.

**4.10 Iron status**

From the stored blood samples, after breaking the code, 200 samples (collected at 10 months of age) will be selected randomly from the placebo group of children from each site for assessing the serum ferritin levels. Measuring serum ferritin levels is needed to assess the background level of iron deficiency in the study population.

**4.11. Evaluation of resistance to study drugs**

Comparison of the relative efficacies of short and long acting drugs would be confounded if there were major differences in the level of parasite resistance to the drugs under investigation. It is likely that both Lapdap and mefloquine will be highly effective in the study area but it is essential to confirm this. Thus, a subsidiary trial of efficacy of the study drugs for clearing asymptomatic parasitaemia will be carried out (see section 4.5). We opted to study the efficacy of the study drugs to clear asymptomatic parasitaemia instead of clinical malaria, because children with asymptomatic parasitaemia are often semi-immune to the parasite strain causing their infection and the parasite density is usually lesser in asymptomatic than in symptomatic infections. Thus different levels of efficacy may be obtained when asymptomatic or symptomatic infections are treated. This will be primarily a descriptive study. It is anticipated that parasite clearance by day 14 will be over 90% in the mefloquine and Lapadap treatment groups but much less in the SP group.

We expect a 10% loss to follow up at 24 months of age and so around 3340 children (low and high transmission area combined) would be screened for this sub-study. We assume that 20% of these children will have parasitaemia and thus 668 children would be allocated to mefloquine, lapdap, or SP treatment group. We assume efficacies of 90% at day 14 in children in the mefloquine and Lapdap groups, and 50% for children in the SP group, the study will have adequate power to estimate the efficacy of these drugs with acceptable level of confidence limits (95% confidence limits of efficacies of mefloquine and lapdap would be +/- 4% and that of SP would be +/- 7%.

Resistance to SP is linked strongly to mutations in the dihdyrofolate reductase (DHFR) and dihydropteroate synthase (DHPS) genes and resistance to Lapdap is probably also strongly related to mutations in these genes, although this has not been established so clearly. There are no definitive molecular markers for mefloquine resistance although some linkage to mutations in the *Pfmdr* gene has been described (24). In order to define the overall level of resistance to anti-folate drugs in the study area, DNA will be extracted from filter paper blood samples obtained from children in the control group who are parasitaemic when blood samples are collected at 10 months of age and tested for DHFR and DHPS mutations. These parasites will not have been subjected to selection by drugs given for IPTi and should reflect the overall pattern of resistance in the community. With a total of around 930 children in the placebo control and an overall parasite prevalence of 25% there should be around 230 samples available for molecular analysis. Information obtained on the background level of resistance to anti-folate drugs will allow comparisons to be made between the results of this trial and other trials of IPTi which have used, or are using, SP.

**4.12. Laboratory procedures**

**Haemoglobin**: capillary blood will be collected in a microcuvette and examined in a Haemocue machine to determine Hb.

**Malaria parasite**: Thick and thin blood smear made from capillary blood will be stained with Giemsa stain and examined under light microscope. To estimate the parasite density, parasites in thick film fields will be counted until 200 leukocytes have been counted an the parasite count will be multiplied by a factor of 40 to give parasites per µl of blood. All blood films will be read twice by individual observers. Discrepant findings will be reviewed by a supervisor and a consensus on positivity and density will be reached. A quality control system will be established to ensure the accuracy of blood film readings and Hb determinations.

**Drug Assays**: Mefloquine, dapsone and pyrimethamine concentrations will be measured in randomly collected urine samples obtained at three day follow-up visits using an HPLC method developed at LSHTM (Kaur, unpublished) which allows the detection of several anti-malarials in one chromatogram. Due to the problems of collecting urine from infants we will use blood spots collected on filter paper from finger or heel prick samples.

**Molecular markers**: DNA will be extracted from filter paper blood samples obtained from parasitaemic children and tested for mutations in DHFR and DHPS genes using standard PCR and SSOP techniques developed at LSHTM.^40^, as well as being tested for mutations in the G6PD gene and haemoglobin genes using a modified SSOP detection method developed at CMP, University of Copenhagen^42^.It is planned to undertake both chromatographic and molecular studies at the new laboratory being built at Bombo Hospital, Tanga on the edge of the study area with support from the Bill and Melinda Gates Foundation. This laboratory should be operational by the end of 2004. Measles antibodies will be measured by a standardised neutralisation assay at a central laboratory contracted by WHO to undertake serology for the consortium.

**Anti-VSA antibody assays**: Anti-VSA antibodies will be measured with a flow cytometric assay that allow the assessment of levels of plasma antibodies to a panel of *in vitro* cultured parasite isolates, some of which are commonly and others rarely recognized. Further antibody assays will be used including ELISA and adhesion-blocking assays. Levels of antibodies and the proportion of parasites recognized will be compared between treatment groups and analysed with respect to clinical protection with statistical regression models. We will compare the level and repertoire of anti VSA antibodies at 10 and 18 months of age, and the incidence of malaria in the second year of life between the study arms. We will include the following terms in a Poisson regression model to assess the effect to IPTi on immunity and clinical malaria: IPTi regime, level of anti VSA antibodies, age, sex, transmission levels and other potential confounders identified during the analysis as predictors and incidence of malaria as the outcome.

**5. Monitoring of the trial**

The trial will be conducted under conditions of good clinical practice (GCP), following as closely as possible the ICH guidelines. Standard operating procedures (SOPs) will be developed for all major operations. A DSMB will be established to look after the trial. Members will include an experienced clinician, a statistician and a person with local knowledge of the study area. Discussions will be held with the DSMB prior to the start of the trial to identify their requirements for adverse events reporting and these will be met. The DSMB will also be consulted about the advisability of an interim analysis, in particular for the SP group as this drug may prove to be ineffective.

At each study site, a local physician will be assigned to be the local safety monitor. He or she will have access to the codes of the study drugs. All serious adverse events will be reported to the local safety monitor by the project physician. In the event of a serious adverse event associated with a study drug, the local safety monitor will be empowered to break the code for that child and, if it is considered necessary to temporarily suspend the trial, prior to discussion with the DSMB.

It is proposed that a clinical monitor will be appointed by the IPTi consortium to monitor all the trials conducted under its auspices and it is anticipated that the monitor will visit the study site on at least one occasion during the trial and provide advice that will help to ensure that the study is conducted to the highest clinical standard.

1. **Data management and analysis**

Data will be entered twice into a database using access software. The accuracy of data input will be checked and validated using a customized validation programme. The comparison of the incidence of malaria and severe malaria will be analysed using Poisson regression models. The statistical significance of differences in mean Hb will be assessed using t-test. We will consider the trials in the low and high transmission areas as separate and we recognise that trials is not adequately powered to make a direct comparison of the effects in the two areas.

There will be two comparisons which are relevant with respect to multiple comparison, Lapdap vs Placebo and SP vs Placebo, and Mefloquine vs placebo will be the primary comparison. As we expect the effects of the two drugs to be similar, it would not be appropriate to power the trial on the Lapdap vs SP comparison. If we make two comparisons at the .025 level of significance then, under the null hypothesis, there is a .05 probability that one will be significant by chance. However we argue it would be appropriate to conduct one-sided tests, on the basis that there is no mechanism by which those given the active drug would have more malaria in the first year than those who do not.

We considered the possibility of powering the study at the 0.025 significance level rather than 0.05 level to allow for an excess of malaria in the first year of life among those on the active drugs in order to get over issue of the multiple comparisons. This would require increasing the sample size by 20%. This would mean increasing the number of infants in the low transmission are from 2200 to 2600 and in the high transmission areas from 1200 to 1440. This would require prolonging the study by four months. Thus,

we have opted to keep the power of the study at 0.05 level for the following reasons: (1) an enhanced incidence of malaria during the first year of life while on an active drug is very unlikely and so we can use a one-sided test (2) enrolling an additional 640 infants would increase substantially the logistical complexity and cost of the study but the added value of this would be small.

A detailed analytical plan will be prepared and submitted to the DSMB before the study drug codes are broken.

1. **Dissemination of results**

The nature of the trial and its possible implications will be discussed with the national Malaria Control and EPI programmes before the start of the trial. On completion of the study, results will be presented to the communities where the trial was undertaken, to local health authorities and to the National Malaria Control programme as well as in the international scientific press.

1. **Ethical considerations**

Giving placebo to infants in one arm of the study is needed for the following reasons: (1) although IPTi with SP has been shown to be beneficial in an area with low transmission, high bednet use, and low SP resistance, there is no clear evidence that IPTi with SP will be beneficial in area with high transmission and high SP resistance; (2) a placebo arm is essential for assessing any interaction between mefloquine and measles vaccine. Study infants will be monitored closely and if they develop clinical malaria they will be identified and treated promptly.

Giving SP to one arm of the study, even though we anticipate that there will be high level of SP resistance in the study area is justified on the grounds that (1) some infants in this arm will benefit from IPTi; (2) as these infants are asymptomatic, there is not imminent danger of progression to clinical malaria. Study infants will be monitored closely and if they develop clinical malaria they will be identified and treated promptly.

Giving mefloquine to one arm of the study is justified as this is the most suitable alternative drug to SP which has a long duration of action and which can be given as a single dose. Mefloquine needs to be used with caution in infants of less than 3 months of age. However, side effects with this drug are rare events and may be balanced by the ability of IPTi with mefloquine to reduce the burden of malaria in infants in areas with SP resistance. Mefloquine is widely used in the private sector for treatment of malaria, including infants, in Tanzania.

Lapdap has been shown to be safe in infants and it is envisaged that this drug will be used widely in Africa in the future.

Collecting blood samples causes minor discomfort. However, the results of this blood examination will be used to offer appropriate treatment. The MoH of Tanzania offers free treatment for children. The project has earmarked a budget for drugs if there is a shortage of drugs for treatment of study children.

Prior to the start of the study the nature of the trial will be discussed with the study population at a series of village meetings. Details of the trial will be discussed with health and civil authorities in the study area. The benefits and risks of taking part in the study will be explained to caretakers at an individual level and their written consent will be obtained before enrolling infants into the study. Approval for the study will be obtained from the ethics committees of Tanzania and the LSHTM

**9. Budget**

The details of the budget are shown in Table 8.

**Equipments:** one four wheel drive vehicle is needed in each site for day to day running of the project at EPI clinics. An additional vehicle is needed for the co-ordinating team. Six motor bikes are needed in each site for home visits for delivering drugs, monitoring adverse events and follow up of defaulters. Two computers are needed in each site for double entry of data and one computer is needed for the central office. Three study health facilities do not have a microscope at the moment and two more microscopes are needed for examining slides at the central point for quality control. All study health centres (12) need a haemacue machine and an additional 2 machines are needed for use during home visits. One digital camera per site is needed for making photo ID cards for study children. Two centrifuges (one for each site) for the separation of plasma and blood cells, two -20 freezers and 7600 eppendorf tubes will be need for the immunology component of the study.

**Personnel**: The project director will be a LSHTM member of staff based in Tanzania. An experienced research fellow is needed for this position in order to ensure a high standard of conduct of the trial. One project physician is needed for each site for running the project and to give clinical support to the health centre staff. Two project nurses and ten field assistants per site are needed for running the EPI clinics and for following up of study children at homes. Two data entry clerks and one data manager per site are needed as the data will entered daily and the list of study infants due for follow up has to be generated every week. The clinical officers at the study health centres will play an important role in passive surveillance. Two laboratory technician will be needed in each site for processing and storage of blood samples.

**Consumables:** The laboratory consumables are estimated on the basis of our experience in an IPTi trial in Ghana.

**Travel and subsistence**: This budget is needed for the LSHTM based PIs and the local PIs and collaborators to visit the study site for monitoring progress of the study and to ensure the high standard of the trial. The budget for post graduate training for the Tanzanian staff is essential for capacity building at the NIMR of Tanzania.

**LSHTM inputs**: The LSHTM will contribute in the order of $ 50,000 per year [ Chris Whitty (10%), Hugh Reyburn (10%), Ilona Carneiro (10%) and Brian Greenwood (5%)].

Table 8

| **Budget estimates for the two sites** | |  |  |  |  |  |
| --- | --- | --- | --- | --- | --- | --- |
| **Items** | | **units** | **unit cost($)** | **total cost ($)** |  |  |
| **Capital** |  |  | year 1 | year 2 | year 3 | total |
| 4 wheel drive vehicle | 3 | 30000 | 90000 |  |  | 90000 |
| motor bikes | 12 | 6000 | 72000 |  |  | 72000 |
| computers | 5 | 2000 | 10000 |  |  | 10000 |
| printers | 2 | 300 | 800 |  |  | 800 |
| microscope | 5 | 2000 | 10000 |  |  | 10000 |
| Haemacue machines | 14 | 1000 | 14000 |  |  | 14000 |
| electonic thermometers | 14 | 20 | 280 |  |  | 280 |
| digital camera | 2 | 500 | 1000 |  |  | 1000 |
| **subtotal** |  |  | **198080** |  |  | **198080** |
|  |  |  |  |  |  | 0 |
| **Personnel** |  | cost per month |  |  |  | 0 |
| project director | 1 | 7000 | 84000 | 88200 | 92610 | 264810 |
| project physicians | 2 | 3000 | 36000 | 37800 | 39690 | 113490 |
| project nurses | 4 | 1200 | 14400 | 15120 | 15876 | 45396 |
| Field assistants | 20 | 4000 | 48000 | 50400 | 52920 | 151320 |
| laboratory technicians | 4 | 1200 | 14400 | 15120 | 15876 | 45396 |
| data managers | 2 | 1200 | 14400 | 15120 | 15876 | 45396 |
| data entry clerks | 4 | 800 | 9600 | 10080 | 10584 | 30264 |
| drivers | 3 | 600 | 7200 | 7560 | 7938 | 22698 |
| clinical officers (honororium) | 10 | 2000 | 24000 | 24000 | 24000 | 74000 |
| local safety monitor | 2 | 100 | 2400 | 2400 | 2400 | 7400 |
| local PIs & collaborators (honororium) |  |  | 8000 | 8000 | 8000 | 24000 |
| Lead PIs salary (20%) |  |  | 16000 | 18000 | 20000 | 54000 |
| **subtotal** |  |  | **278400** | **291800** | **305770** | **875970** |
|  |  |  |  |  |  |  |
| **consumables** |  |  |  |  |  |  |
| microcuvettes (100 per box) | 200 | 40000 | 10000 | 20000 | 10000 | 40000 |
| lancets (100 per box) | 400 | 4000 | 1000 | 2000 | 1000 | 4000 |
| glass slides (50 per box) | 400 | 2000 | 500 | 1000 | 500 | 2000 |
| filter papers (boxes of filter mats) | 5 | 1000 | 1000 | 0 | 0 | 1000 |
| stains and lab consumables |  |  | 1000 | 1000 | 1000 | 3000 |
| stationary |  |  | 3000 | 2000 | 2000 | 7000 |
| drugs |  |  | 20000 | 5000 | 5000 | 30000 |
| lab charges for HPLC & SSOP |  |  |  |  | 10000 | 10000 |
| lab charges for ferritin assays |  |  |  |  | 10000 | 10000 |
| lamination of idcards |  |  | 2000 | 2000 | 1000 | 5000 |
| fuel & maintanence of vehicles |  |  | 35000 | 35000 | 35000 | 105000 |
| **subtotal** |  |  | **71500** | **66000** | **64500** | **202000** |
| **travel and subsistance & capacity building** |  |  |  |  |  |  |
| international travel |  |  | 10000 | 5000 | 10000 | 25000 |
| local travel |  |  | 5000 | 2000 | 2000 | 9000 |
| post graduate training for staff |  |  |  | 20000 | 20000 | 40000 |
| **subtotal** |  |  | **15000** | **27000** | **32000** | **74000** |
| **admistration** |  |  |  |  |  |  |
| office rents & communication |  |  | 8000 | 8500 | 9000 | 25500 |
| Research permits and bank charges |  |  | 3000 | 1000 | 1000 | 5000 |
| local collaborators' admin cost |  |  | 14400 | 15900 | 16000 | 46300 |
| **subtotal** |  |  | **25400** | **25400** | **26000** | **76800** |
| **overheads (10% of non-capital expenses)** |  |  | **38490** | **39130** | **41827** | **119447** |
| **grand total** |  |  | **621870** | **404976** | **449330** | **1551297** |

**10. Timetable**

The time schedule of key activities is shown in Table 9.

**Table 9**: **Time Table of activities**

|  | Year 1 (July 2004-June 2005) | | | | Year 2(July 2005- June 2006) | | | | Year 3(July 2006-June 2007) | | | |
| --- | --- | --- | --- | --- | --- | --- | --- | --- | --- | --- | --- | --- |
| Activities | Q1 | Q2 | Q3 | Q4 | Q1 | Q2 | Q3 | Q4 | Q1 | Q2 | Q3 | Q4 |
| Recruitment of staff and preparation of logistics & supplies | X |  |  |  |  |  |  |  |  |  |  |  |
| Sensitisation of the community and launch of the study | X |  |  |  |  |  |  |  |  |  |  |  |
| Enrolment of subjects for the drug sensitivity study |  |  |  |  |  | X |  |  |  |  |  |  |
| Follow up of subjects enrolled in the drug sensitivity study |  |  |  |  |  | X |  |  |  |  |  |  |
| Enrolment of study children |  | X | X | X | X | X |  |  |  |  |  |  |
| Administration of IPTi drugs |  | X | X | X | X | X | X | X | X |  |  |  |
| Passive surveillance of malaria and anaemia |  | X | X | X | X | X | X | X | X | X | X |  |
| Data management |  | X | X | X | X | X | X | X | X | X | X |  |
| Interim data analysis |  |  |  |  |  |  | X |  |  |  |  |  |
| Final analysis & dissemination |  |  |  |  |  |  |  |  |  |  |  | X |

**11. References**

1. Murphy SC, Breman JG. Gaps in the childhood malaria burden in Africa: cerebral malaria, neurological sequelae, anaemia, respiratory distress, hypoglycaemia and complications of pregnancy. Am J Trop Med Hyg 2001; 64 (1-2 suppl) 57-67.
2. WHO. Investing in health research and development. TDR 1996/Gen/96.1.
3. DeMaeyer E, Adiels-Tegman M. The prevalence of anaemia in the world. World Health Stat Q 1985; 38:302-16.
4. Lozoff B, Jimenez E, Wolf AW. Long-term developmental outcome of infants with iron deficiency. New Engl J Med 1991; 325:687-94.
5. Smith A, Hendrickse R, Harrison C, Hayes R, Greenwood B. Iron deficiency anaemia and its response to oral iron: report of a study in rural Gambian children treated at home by their mothers. Ann Trop Peadiatr 1989; 9:6-16.
6. Menendez C, Kahigwa E, Hirt R et al. Randomised placebo controlled trial of iron supplementation and malaria chemoprophylaxis for prevention of sever anaemia and malaria in Tanzanian infants. Lancet 1997; 350:844-850.
7. Oppenheimer S. Gibson F, Macfarlane S et al. Iron supplementation increases prevalence and effects of malaria: report on clinical studies in Papua New Guinea. Trans R Soc Trop Med Hyg 1986; 8):603-612.
8. Kitua A, Smith T, Alosno P et al. The role of low level plasmodium Falciparum parasitaemia in anaemia among infants living in an area of intense and perennial transmission. Trop Med Int Health 1997; 2:325-333.
9. Snow R, Omumbo J, Lowe B et al. Relation between severe malaria morbidity in children and level of plasmodium falciparum transmission in Africa. Lancet 1997; 349:1650-54.
10. Geerlings P, Brabin B, Eggelte T. Analysis of the effects of malaria chemoprophylaxis in children on haematological responses, morbidity and mortality. Bull Wld Hlth Org 2003; 81: 205-216.
11. Bradley-Moore A, Greenwood B, Bradley A et al. Malaria chemoprophylaxis with chloroquine in young Nigerian children. Its effect on haemoglobin measurements. Annals of Tropical Medicine and parasitology 1985; 29:585-595.
12. Greenwood B, David P, Otoo-Forbes L et al. Mortality and morbidity from malaria after stopping malaria chemoprophylaxis, Trans R Soc Trop Med Hyg 1995; 89: 629-633.
13. Schellenberg D, Menendez C, Kahigwa E. et al. Intermittent treatment for malaria and anaemia control at time of routine vaccinations in Tanzanian infants: a randomised, placebo-controlled trial. Lancet 2001; 357:1471-77.
14. Massaga J, Kitua A, Lemnge M et al. Intermittent treatment with amodiaquine markedly reduces the incidence of anaemia and malaria fevers during the first year of life: a randomised placebo controlled trial in an area highly endemic for drug resistant malaria. Abstract number 161. 3^rd^ Pan African Conference on Malaria, 2002, Arusha, Tanzania.
15. Owusu A, Fryauff D, Chandramohan D et. Characteristics of severe anaemia and its association with malaria in young children of the Kassena-Nankana district of northern Ghana. Am J Trop Med Hyg 2002; 67: 371-377.
16. Chandramohan D, Jaffar S, Greenwood BM. Use of clinical algorithms for diagnosing malaria. Trop Med Int Health 2002; 7:45-52.
17. Roper C, Pearce R, Bredenkamp B et al. Antifolate antimalarial resistance in southeast Africa: a population-based analysis. Lancet 2003; 361: 1174-81.
18. Mutabingwa T, Nzila A, Mberu E, Nduaiti E, Winstanley P, Hills E, Watkins W. Chlorproguanil-dapsone for treatment of drug-resistant falciparum malaria in Tanzania. Lancet 2001;358:1218-1223.
19. Schellenberg D, Kahigwa E, Drakeley C, Malende A, Wigayi J, Msokame C, Aponte JJ, Tanner M, Mashinda H, Menendez C, Alonso PL. The safety and efficacy of sulphadoxine-pyrimethamine, amodiaquine and their combination in the treatment of uncomplicated *Plasmodium falciparum* malaria. Amer J Trop Med Hyg 2002 ;67:17-23.
20. Bredenkamp B, Sharp B, Mthembu S, Durrheim D, Barnes K. Failure of sulphadoxine-pyremethamine in treating plasmodium falciparum malaria in Kwazulu-Natal. S Afr Med J 2001; 91:970-72.
21. Olliaro P, Nevill C, LeBras J, Ringwald P, Mussano P, Garner P, Brasseur P. Systematic review of amodiaquine treatment in uncomplicated malaria. Lancet 1996;348:1196-1201.
22. Adjuik M, Babiker A, Borrman S, Brasseur P, Cisse M, Diallo S, Faucher JF, Garner P, Gikunda S, Kremsner PG, Krishna S, Lell B, Loolpapit M, Matsiegui P-B, Missinou MA, Mwanza J, Ntoumi F, Olliaro P, Osimbo P, Rezbach P, Some E, Taylor WR. Amodiaquine-artesunate versus amodiaquine for uncomplicated, *Plasmodium falciparum* malaria in African children: a randomised, multicentre trial. Lancet 2002;359:1365-1372.
23. Staedke SG, Kamya MR, Dorsey G, Gasasira A, Ndeezi G, Charlebois ED, Rosenthal PJ. Amodiaquine, sulfadoxine/pyrimethamine, and combination therapy for treatment of uncomplicated falciparum malaria in Kampala, Uganda: a randomised trial. Lancet 2001;358:368-374.
24. Sowunmi A, Oduola A,. Open comparison of mefloquine, sulfadoxine/pyrimethamine, and chloroquine in acute uncomplicated falciparum malaria in children. Trans R Soc Trop Med Hyg 1995; 89:303-305.
25. Mockenhaupt FP. Mefloquine resistance in *Plasmodium falciparum*. Parasitol Today 1995;11:248-253
26. MacArthur JR, Stennies GM, Macheso A, Kolczak MS, Green MD, Ali D, Barat LM, Kazembe PN, Ruebush TK. Efficacy of mefloquine and sulphadoxine-pyrimethamine for the treatment of uncomplicated *Plasmodium falciparum* infection in the Machinga District, Malawi, 1998. Am J Trop Med Hyg 2001;65:679-684.
27. World Health Organization. The use of antimalarial drugs. WHO/CDS/RBM/2001.33.
28. Luxemburger C, Price R, Nosten F, Ter Kuile F, Chongsuphajaisiddhi, White N. mefloquine in infants and young children. Annals of Tropical Paediatrics 1996; 16: 281-286.
29. Kanyok T Clinical development of Lapdap for malaria. Presentation at a meeting of the American Society of Tropical Medicine and Hygiene, Atlanta, November 12 th, 2001.
30. Sulo J, Chimpeni P, Hatcher J, Kublin JG, Plowe CV, Molyneux ME, Marsh K, Taylor TE, Watkins WM, Winstanley PA. Chloroproguanil-dapsone versus sulfadoxine-pyrimethamine for sequential episodes of uncomplicated falciparum malaria in Kenya and Malawi: a randomised clinical trial. Lancet 2002;360:1136-1143.
31. Trigg JK, Mbwana H, Chambo O, Hills E, Watkins W, Curtis CF. Resistance to pyrimethamine-sulfadoxine in *Plasmodium falciparum* malaria in 12 villages in Northeast Tanzania and a test of chlorproguanil dapsone. Acta Tropica 1997;63:185-89.
32. Nosten F, Brasseur P. Combination therapy for malaria. Drugs 2002;62:1315-1329.
33. Marsh K, Otoo L, Hayes RJ, Carson DC, Greenwood BM. Antibodies to blood antigens of *Plasmodium falciparum* in rural Gambians and their relation to protection against infection. Trans R Soc Trop Med Hyg 1989; 83: 293-303.
34. Kyes S, Horrocks P, Newbold C. Antigenic variation at the infected red cell surface in malaria. Annu Rev Microbiol 2001; 55: 673-707.
35. Bull PC, Lowe BS, Kortok M, Marsh K. Antibody recognition of *Plasmodium falciparum* erythrocyte surface antigens in Kenya: evidence for rare and prevalent variants. Infect Immun 1999; 67: 733-739.
36. Bull PC, Kortok M, Kai O, Ndungu F, Ross A, Lowe BS, Newbold CI, Marsh K. *Plasmodium falciparum*-infected erythrocytes: agglutination by diverse Kenya plasma is associated with severe disease and young host age. J Infect Dis 2000; 182: 252-259.
37. Nielsen M, Staalsoe T, Kurtzhals J, Goka BQ, Dodoo D, Alifrangis M, Theander TG, Akanmori BD, Hviid L. *Plasmodium falciparum* variant surface antigen expression varies between isolates causing severe and non-severe malaria and is modified by acquired immunity. J Immunol 2002; 168: 3444-3450.
38. Ofori M., Dodoo D, Staalsoe T, Kurtzhals J, Koram K, Theander TG, Akanmori BD, Hviid L. Malaria-induced acquisition of antibodies to Plasmodium falciparum variant surface antigens. Infect Immun 2002; 70: 2982-2988.
39. Msuya S, Mbivzo E, Stray-Pedersen B et al. Reproductive tract infection and the risk of HIV among women in Moshi, Tanzania. Acta Obstet Gynecol Scand 2002; 81: 886-893.
40. Pearce R, Drakeley C, Chandramohan D, Mosah F, Roper C. Molecular determination of point mutation haplotypes in the dihydrofolate reductase and dihydropteroate synthetase of *Plasmodium facliparum* in three districts of northern Tanzania. Antimicrobial Agents and Chemotherapy 2003; 47: 1347-1354.
41. Staalsoe T, Giha HA, Dodoo D, Theander TG, Hviid L. Detection of antibodies to variant antigens on *Plasmodium falciparum-*infected erythrocytes by flow cytometry. Cytometry 1999; 35: 329-336.
42. Alifrangis M, Enosse S, Pearce R, Drakeley C, Roper C, Khalil IF, Nkya WM, Rønn AM, Theander TG and Bygbjerg IC. A simple, high-throughput method to detect *Plasmodium falciparum* single nucleotide polymorphisms in dihydrofolate reductase, dihydropteroate synthase, and *P. falciparum* chloroquine resistence genes using polymerase chain reaction and enzyme-linked immunosorbent assay-based technology. Am J Trop Med Hyg *72:* 155-162, 2005
43. Ruwende C, Hill A. Glucose-6-phosphate dehydrogenase deficiency and malaria..
     J Mol Med. 1998 Jul;76(8):581-8.
44. Lell B, May J, Schmidt-Ott RJ, Lehman LG, Luckner D, Greve B, Matousek P, Schmid D, Herbich K, Mockenhaupt FP, Meyer CG, Bienzle U, Kremsner PG. The role of red blood cell polymorphisms in resistance and susceptibility to malaria.
    Clin Infect Dis. 1999 Apr;28(4):794-9.
45. Alloueche A, Bailey W, Barton S, Bwika J, Chimpeni P, Falade CO, Fehintola FA, Horton J, Jaffar S, Kanyok T, Kremsner PG, Kublin JG, Lang T, Missinou MA, Mkandala C, Oduola AM, Premji Z, Robertson L, Sowunmi A, Ward SA, Winstanley PA. Comparison of chlorproguanil-dapsone with sulfadoxine-pyrimethamine for the treatment of uncomplicated falciparum malaria in young African children: double-blind randomised controlled trial. Lancet. 2004 Jun 5;363(9424):1843-8

12 **Appendix 1**

The incidence of severe anaemia over the first year of life may be modelled using a deterministic compartmental model (see Figure above), with the flow between epidemiological states described by a set of linked, first order differential equations. The model is flexible, and may be modified (e.g. with additional epidemiological classes added) to better fit our biological understanding of the system.

In its simplest form, the model follows a cohort of children with a life expectancy at birth of 1/δ years, where δ is the underlying annual mortality rate. By a given age, all surviving cohort members are considered to be free from maternal antibodies and so susceptible to malaria infection (S_0_). These children acquire infection at an age-independent force of infection, λ (such that 1/ λ is the average age of first infection), thus becoming infected for the first time (I_1_). Once infected, they may recover (naturally or through treatment) at a rate β_1_ or develop severe anaemia at a rate α_1_. Those that recover move into the S_1_ class (susceptible, but having been infected once before). Those that develop severe anaemia for the first time (A_1_) die at a rate ε_1_ additional to the underlying mortality rate δ. Those that survive move into the S_1_ category at a rate v_1_. Individuals in the S_1_ acquire their second infection at rate λ, enter the class I_2_ and, as before, may recover to the S_2_ category (susceptible but with two previous infections) at rate β_2_ or develop severe anaemia at rate α_2_, in which case they are subject to an additional ε_s_ mortality rate. The model may be extended indefinitely, tracking the number of infections the cohort experiences. In the above example, the model is closed after three infections, by having recovery from the I_3_ and A_3_ categories to the S_2_ susceptible class (i.e. susceptible, but having been infected two times or more).

This model structure allows us to vary the rate at which individuals develop severe anaemia (α_i_) or recovery without severe disease (β_i_) as a function of the number of previous infections (i) experienced by the child. In this way, we are able to model the effects of acquired immunity in reducing the likelihood of an infected child developing severe anaemia. The model may also be extended to include variations in the parameters α_i_ and β_i_ as a function of age of the child, as well as infection history.

The effects of IPTi may be modelled by removing to a chemoprophylatically protected state, C, (not shown in above Figure) a fixed proportion, Ω, of all the epidemiological classes at designated times, T, over the first year of life. Individuals move from the C class back into the appropriate S_i_ categories at a rate π, where 1/π is the average duration of chemoprophylaxis resulting from IPTi. The model, therefore, allows us to quantify the expected reduction in severe anaemia incidence, under different IPTi strategies (e.g. varying T and Ω) in different epidemiological setting (e.g. by varying λ). The model can also be extended to include cerebral malarial, as well as severe anaemia, beyond the first year of life.

**Appendix 2**

**A comparative study of the efficacy of two or three doses of anti-malarial for IPTi in low transmission areas**

All IPTi studies currently under way utilise a schedule of drug administration which is very similar to that employed in the initial trial in Ifakara, Tanzania (13) with administration of the anti-malarial at the time of second DPT, third DPT and measles vaccinations, corresponding to the ages of approximately 3, 4 and 9 months. However, in low transmission areas relatively few infants will be infected with malaria before the age of about 6 months, in part because they are kept protected from exposure. In such situations, it is uncertain whether the schedule of drug administration for IPTi currently being advocated is appropriate; in such situations a two dose schedule with one dose given at the age of 4 months with DPT3 and the other at the age of 9 months with measles vaccine might be as effective as a three dose schedule, cheaper and safer.

For these reasons we propose a study in the South Pare district in which the efficacy of two and three dose regimens of the drug shown to be most effective in the present trial are compared. This would be an equivalence study with the incidence of clinical malaria during the first year of life and anaemia at the age of nine months as the main trial end-points. The study design would be similar to the one proposed in this submission and would use the same study team. However, until the first study has been completed, it is not known which would be the most appropriate drug to use for this study. Thus, investigation of this important issue has not been included in the present proposal, or budgeted for, but it is proposed as a potentially valuable continuation of investigations into the most effective ways of applying IPTi.

**APPENDIX 3**

**Adverse Events**

**DRAFT- to be agreed with the DSMB**

**Eliciting and Documenting Adverse Experience**

It is the responsibility of the investigator to document all adverse events or reactions which occur during the investigation. An adverse experience includes any noxious, pathological or unintended change in anatomical, physiological or metabolic functions as indicated by physical signs, symptoms and/or laboratory changes occurring in any phase of the clinical study whether associated with the study drug or placebo and whether or not considered drug related. This includes an exacerbation of pre-existing conditions or events, inter-current illnesses, drug interaction or the significant worsening of the disease under investigation that is not recorded elsewhere in the case record form under specific efficacy assessments. Anticipated day-to-day fluctuations of pre-existing conditions, including the disease under study that do not represent a clinically significant exacerbation or worsening need not be considered adverse events or reactions.

In the case of studies involving a marketed drug in an established indication, an adverse experience includes significant failure of expected pharmacological or biological action.

All adverse events or reactions occurring after the start of the study **MUST BE REPORTED AT THE EARLIEST POSSIBLE OPPORTUNITY TO THE TRIAL COORDINATOR AND THE TRIAL CLINICIAN.**

At each visit/assessment, adverse events or reactions will be evaluated by the investigator. Adverse events or reactions not previously documented in the study will be recorded in the adverse experience section of the subject's case record form. The nature of each experience, date and time (where appropriate) of onset, duration, severity and relationship to treatment should be established. Details of changes to the dosage schedule or any corrective treatment should be recorded on the appropriate pages of the case record form.

Adverse events or reactions already documented in the CRF i.e. at a previous assessment and designated as 'continuing' should be reviewed. If these have resolved, the documentation in the CRF should be completed. NB. If an adverse experience changes in frequency or severity during a study period, a new record of the experience will be started.

**Assessment of Severity**

The most severe stage of the adverse reaction should be assigned to one of the following categories:

Mild: For example, an adverse experience which is easily tolerated by the subject, causing minimal discomfort not interfering with everyday activities, e.g. vomiting.

Moderate: For example, an adverse experience which is sufficiently discomforting to interfere with normal everyday activities e.g. headache.

Severe: For example, an adverse experience which prevents normal everyday activities e.g. necrotizing dermatitis (Stephen- Johnson syndrome).

**Assessment of Causality**

Every effort should be made by the investigator to explain each adverse experience and assess its relationship, if any, to study drug treatment. Causality should be assessed using the following categories: not related, unlikely, possible, probable, most probable, insufficient data.

The degree of certainty with which an adverse experience is attributed to drug treatment (or alternative causes, e.g. natural history of the underlying diseases, concomitant therapy, etc.) will be determined by how well the experience can be understood in terms of one or more of the following:

· Known pharmacology of the drug

· Reaction of similar nature being previously observed with this drug or class of drug

· The experience having often been reported in the literature for similar drugs as drug related e.g. skin rashes, blood dyscrasia

· The experience being related by time to drug ingestion terminating with drug withdrawal (dechallenge) or reproduced on rechallenge

**Following-up of Adverse Events**

Investigators should follow-up subjects with adverse experiences until the event has subsided (disappeared) or until the condition has stabilised.

**Serious Adverse Events**

**Definition of Serious Adverse Events**

A serious adverse experience is any event which is fatal, life threatening, disabling or incapacitating or results in hospitalisation, prolongs a hospital stay or is associated with congenital abnormality, cancer or overdose (either accidental or intentional). In addition any experience which the investigator regards as serious or which would suggest any significant hazard, contraindication, side effect or precaution that may be associated with the use of the drug should be reported as a serious event.

**Life threatening** - An adverse experience is life threatening if the subject was at immediate risk of death from the event as it occurred; i.e. it does not include a reaction that if it had occurred in a more serious form might have caused death. For example, drug induced hepatitis that resolved without evidence of hepatic failure would not be considered life threatening even though drug induced hepatitis can be fatal.

**Disability/incapacitating**:- An adverse experience is incapacitating or disabling if the experience results in a substantial and/or permanent disruption of the subject's ability to carry out normal life functions.

**Appendix 4 – Blister pack of drugs for IPTi course 1 & 2**

**SP Arm MQ Arm LAPDAP Arm Placebo Arm**

**Day 1**

**Day 2**

**Day 3`**

**SP = Sulphadoxine-Pyrimethamine**

**SP-P = Sulphadoxine-Pyrimethamine Placebo**

**MQ = Mefloquine**

**CD = Chlorproguanil-Dapsone (Lapdap)**

**CD-P = Chlorproguanil-Dapsone Placebo**

**Appendix 5 – Blister pack of drugs for IPTi course 3**

**SP Arm MQ Arm LAPDAP Arm Placebo Arm**

**Day 1**

**Day 2**

**Day 3`**

**SP = Sulphadoxine-Pyrimethamine**

**SP-P = Sulphadoxine-Pyrimethamine Placebo**

**MQ = Mefloquine**

**CD = Chlorproguanil-Dapsone (Lapdap)**

**CD-P = Chlorproguanil-Dapsone Placebo**
